# Automated ROI detection allows rapid quantification of synaptic activity across tens of thousands of synapses in cell culture

**DOI:** 10.3389/fnsyn.2026.1832103

**Published:** 2026-05-28

**Authors:** John Carl Begley, Harald Prüss, Paul Turko, Camin Dean

**Affiliations:** 1 Institute of Biology, Humboldt-Universität zu Berlin, Berlin, Germany; 2 German Center for Neurodegenerative Diseases (DZNE) Berlin, Berlin, Germany; 3 Einstein Center for Neurosciences Berlin, Berlin, Germany; 4 Shared Primary Neuron Facility (SPNF), Institute for Integrative Neuroanatomy, Charité – Universitätsmedizin, Berlin, Germany; 5 Department of Neurology and Experimental Neurology, Charité – Universitätsmedizin Berlin, Berlin, Germany; 6 Bernstein Center for Computational Neuroscience Berlin, Berlin, Germany

**Keywords:** calcium imaging, NMDAR auto-antibodies, automated ROI detection, presynaptic function, post-synaptic function

## Abstract

Synapses are the basic unit of information transfer between neurons. Their dysfunction is a common trigger of cognitive diseases and disorders. However, high-throughput analysis methods to assess synaptic function and dysfunction are lacking. Calcium imaging in cultured neurons in the absence of Mg^2+^ and presence of TTX allows visualization of NMDAR-dependent spontaneous synaptic calcium transients, which report pre and postsynaptic function. Here, we introduce a high-throughput automated analysis pipeline that combines Suite2p ROI detection and Python scripts to analyze tens of thousands of synapses and quantify changes in presynaptic vesicle fusion rates (frequency), postsynaptic function (amplitude), and the number of functional synapses. We use this pipeline to test known NMDAR agonists (glycine) and antagonists (ketamine, memantine, APV), presynaptic function modulating compounds (PDBu), and encephalitis patient-derived NMDAR auto-antibodies, where our pipeline proved more sensitive in detecting dysfunction at the single-synapse level than other methods. The ability to detect, track, and quantify activity across tens of thousands of synapses and millions of synaptic calcium transients using this pipeline will aid drug discovery of compounds that protect synapse function.

## Introduction

Calcium imaging is a well-established method for studying neuronal activity and connectivity. It is routinely used to infer electrical activity across large populations of neurons *in vivo* and *in vitro* in place of, or alongside, more invasive and low-throughput electrophysiological recordings. The improved sensitivity of genetically-encoded calcium indicators allows the detection of subthreshold changes in intracellular calcium concentrations in neurons and their processes (Li et al., 2019). Synaptic calcium imaging can be achieved by imaging in a modified artificial cerebrospinal fluid (ACSF) solution that contains tetrodotoxin (TTX), to block action potentials, and 0 mM Mg^2+^, to remove the magnesium block of NMDA receptors (NMDARs). This allows NMDARs in dendritic spines to report spontaneous glutamate release from presynaptic vesicles by calcium influx into postsynaptic dendrites.

The combination of 0 Mg^2+^ and TTX was initially used in electrophysiological experiments to demonstrate that NMDARs are the primary glutamatergic receptors to flux calcium (Iino et al., 1990; Mayer et al., 1984). Removing magnesium to ungate NMDARs was then co-opted for calcium imaging to study synaptic calcium transients *in vivo* in response to glutamate uncaging (Sobczyk et al., 2005). This method was later adapted to visualize synaptic calcium transients *in vitro* (Andreae and Burrone, 2015). Synaptic calcium imaging has been applied *in vitro* to investigate NMDAR trafficking and activity in developing dendrites (Andreae and Burrone, 2015), the effect of beta amyloid (Prikhodko et al., 2024; Sinnen et al., 2016), and the effect of a patient-derived NMDAR autoantibody (Dean et al., 2022) on synaptic calcium transients, for example.

Despite the adoption of this method by multiple research groups, the analysis of synaptic calcium imaging recordings has remained low-throughput. To the best of our knowledge, all previous uses of this method have relied on either manual or semi-manual approaches to identify potential synaptic regions of interest (ROIs) in combination with custom-written code and software for analysis. Other publications analyzing spontaneous synaptic calcium transients used FIJI for manual ROI detection, Clampex for extraction of fluorescence signal and MATLAB for analysis (Dean et al., 2022; Metzbower et al., 2019), FIJI for manual ROI detection and MetaMorph for extraction of fluorescence signal and analysis (Sinnen et al., 2016; Prikhodko et al., 2024), or manual ROI detection and MATLAB analysis in combination with MiniAnalysis software from Synaptosoft (Andreae and Burrone, 2015; Walker et al., 2017). These manual methods are not only time- and labor-intensive but also susceptible to user bias and may potentially miss small, nuanced changes in synaptic activity due to small sample sizes. Manual identification of ROIs is also incompatible with larger datasets, leaving previous studies to rely on, at most, datasets of 1,000–2,000 synapses per experimental condition. Most neurons form thousands of synapses, so there is clear room for optimization. Without the limitations of manual detection and low-throughput analysis, this method could be used for large-scale screenings of compounds and their effects on synapses by reporting functional synapse number, presynaptic vesicle fusion rates and neurotransmitter release properties, and postsynaptic receptor function.

We therefore sought to automate the detection and analysis of synaptic calcium events with a single open-source pipeline that would allow all visible synapses to be detected and analyzed with minimal user intervention. Many existing somatic calcium imaging analysis tools could potentially be adapted to analyze synaptic calcium imaging data at scale, including EZCalcium (Cantu et al., 2020), FluoroSNNAP (Patel et al., 2015), CaImAn (Giovannucci et al., 2019), SIMA (Kaifosh et al., 2014), SICT (Mancini et al., 2018), LC_Pro (Francis et al., 2014) and Suite2P (Pachitariu et al., 2016). One synapse-specific analysis tool exists, SynActJ (Schmied et al., 2021), however, it was built to identify evoked synaptic activity and requires input of stimulation time for comparison of frames before and after. We eliminated MATLAB-based approaches (FluoroSNNAP, EZCalcium, SICT) since they are not open-source. LC_Pro was also eliminated because it requires both FIJI and R for analysis, and manual creation of output directories and inspection of videos to identify appropriate ROI size, and does not offer motion correction or image registration. Alternative Python-based approaches were either difficult to install and navigate for someone unfamiliar with coding (CaImAn), or outdated (SIMA; Python v2.7). Suite2p was selected as the best platform to adapt based on its ability to (1) detect and process thousands of individual ROIs quickly, (2) handle a variety of image types (e.g., tif, nd2, mp4, binary, sbx, dcimg), and (3) perform motion correction and fluorescence extraction as backend components, allowing image registration and ROI detection to be performed automatically. Although it is possible to recreate these same functions in FIJI or CellProfiler, a secondary coding language (e.g., R, Python, MATLAB) would be required for post-processing to calculate frequency, amplitude, and synapse number (after filtering). Since Suite2p is Python-based, post-processing can be performed in the same software. In addition, Suite2p offers a user-friendly installation guide, intuitive documentation and interactive graphical user interface (GUI) for visualizing data and troubleshooting detection errors, which is particularly useful for users trying coding or calcium imaging for the first time.

We adapted Suite2p to detect synaptic ROIs. Then, we developed and validated an automated workflow in Python combining Suite2p ROI detection and analysis of spontaneous synaptic calcium transients in neuronal cultures expressing GCaMP6f. Using these scripts, we were able to identify synaptic calcium transient sites and calculate frequency and amplitude at individual synapses, and active synapse numbers. This allowed us to curate datasets with tens of thousands of individual synapses. From these datasets, we could investigate changes to individual synapses and populations of synapses before, during, or after treatment with potential synapse-modulating compounds.

With this analysis pipeline, we reliably report NMDAR-dependent synaptic calcium transients, benchmarked by known agonists (glycine) and antagonists (APV), and detect changes in presynaptic release (following application of phorbol esters). We measure responses to ketamine—currently being investigated for treatment of depression (Berman et al., 2000; Raza et al., 2025), PTSD (Krystal et al., 2017; Valizadeh et al., 2025), acute pain (Gurnani et al., 1996; Trevino et al., 2025), and chronic pain (Carr et al., 2004; Tankha et al., 2025)—and memantine, a preferential antagonist of extrasynaptic NMDARs developed to mitigate the symptoms of advanced Alzheimer’s disease and dementia (Ditzler, 1991) that is now in clinical trials for treatment of Parkinson’s Disease (Jeong and Lee, 2025). We also report effects of and differences between encephalitis patient-derived NMDAR auto-antibodies. By quantifying the function of all active synapses automatically, this pipeline reliably captures a variety of possible effects of compounds on synaptic function.

## Methods

### Cell culture preparation

Primary corticohippocampal cultures were prepared under sterile conditions from postnatal day 0–2 (P0–P2) wild-type Wistar rat pups using protocols adapted from Turko et al. (2019). A total of 12 litters of rat pups were used (3 for acute treatments with glycine, NBQX, ketamine, memantine, and DMSO vehicle, 4 for anti-NMDAR patient autoantibodies, and 5 for ketamine, memantine, and DMSO vehicle overnight treatments). Isolated tissue was rapidly dissected and transferred into ice-cold (4 °C) cell culture buffer (116 mM NaCl, 5.4 mM KCl, 26 mM NaHCO_3_, 1.3 mM NaH_2_PO_4_, 1 mM MgSO_4_•7H_2_O, 1 mM CaCl_2_•2H_2_O, 0.5 mM EDTA•2Na•2H_2_O, and 25 mM D-glucose, pH = 7.4). Tissue was diced and then incubated at 37 °C in 5 mL cell culture buffer containing 1.5 mg/mL Papain (Sigma) for 25 min. The digested tissue was then triturated with a fine-tip Pasteur pipette in 3 separate 15 mL Falcon tubes, each containing 4 mL cell culture buffer supplemented with 10 mg/mL bovine serum albumin (BSA; Sigma). The triturated tissue was then combined into a single 12 mL suspension and centrifuged at 3000 RPM for 3 min. The supernatant was discarded, and the pellet resuspended in pre-warmed (37 °C) complete Neurobasal-A medium (complete-NBA) without phenol red (Gibco) supplemented with B27 (1×), GlutaMAX (1×), and Penicillin–Streptomycin (100 U/mL; all from Gibco). Cells were then passed through a 30 μm cell strainer (Partec CellTrics; Sysmex). To determine the number of viable cells in suspension, cells were stained with trypan blue and counted on a hemocytometer. Cells were plated at 50,000 cells per well, in 24-well glass-bottom plates with high-performance #1.5H cover glass (0.170 ± 0.005 mm, *In Vitro* Scientific), in 500 μL of complete-NBA medium without phenol red.

### Viral transduction

Diluted cell suspensions (1.25 million cells) were treated individually with 1 μL of AAV2/1 human synapsin promoter-driven GCaMP6f (2.0 × 10^13^ v.g./mL, pAAV. Syn.GCaMP6f.WPRE.SV40, Addgene; Chen et al., 2013) and vortexed. Cells were seeded at a density of 50,000 cells in 500 μL complete-NBA medium without phenol red/well (with ~0.05 μL of virus per well) on poly-L-lysine-coated 24-well glass-bottom plates. Immediately after plating, cultures were allowed to equilibrate to the incubator environment (humidified, 5% CO_2_ at 37 °C) in plastic containers, which were then sealed and maintained in the incubator until experiments were performed. We found that these sealable containers minimize the ‘edge-effect’ (where the health and density of cells in wells at the edge of the culture plate may be compromised compared to those in the center) by slowing the exchange of CO_2_ and humidity between the plates and the incubator, thereby reducing environmental fluctuations caused by repeated opening of the incubator door throughout the day.

### Synaptic calcium imaging

For synaptic calcium imaging, DIV19-22 rat corticohippocampal cultures were imaged without magnesium (0 mM Mg^2+^) and in the presence of 1 μM tetrodotoxin (TTX) in ACSF (140 mM NaCl, 2.5 mM KCl, 10 mM Glucose, 10 mM HEPES, and 2 mM CaCl_2,_ pH adjusted to 7.4 with NaOH). For overnight treatments, pharmacological agents or antibodies were added to cultures the day before imaging and remained in cultures until media were exchanged for 1 mL of 0 mM Mg^2+^ ACSF with TTX immediately before synapse calcium imaging the next day. For acute treatments, wells were washed once with 0.5 mL of 0 mM Mg^2+^ ACSF with TTX and imaged in 0.5 mL of the same media. Acute treatments were then bath applied in an additional 0.5 mL of 0 Mg^2+^ solution with TTX containing 2× concentrated compound, to promote rapid mixing to the desired final concentration.

Cultures were imaged using a Nikon Ti-2 Eclipse widefield inverted fluorescence microscope with an incubation chamber at 32 °C and a 40× water-immersion objective (NA 1.25), and 35 ms triggered LED exposure using 20–25% LED power. Calcium transients were recorded at 20 frames per second (fps), i.e., 50 ms interval between images (512 × 512 or 596 × 596 pixels in size; 0.325 μm/pixel) acquired using an sCMOS PCO edge camera (Excelitas). During image acquisition, 2 × 2 binning was applied to recordings to reduce file size. Regions imaged were manually selected based on neuronal viability, morphological integrity, and visible synaptic calcium transients; these criteria were applied consistently across all conditions. Regions with 1–4 neuronal somata were chosen. A maximum of 9 regions could be automatically imaged in sequence at defined coordinates before the water for immersion had to be replenished. For overnight application of compounds, regions of interest in control or treated conditions were imaged for 3 min each. For acute bath application of compounds, regions of interest were imaged for 3 min at baseline, pharmacological agents were applied, and the same regions were imaged again for 3 min. All compounds tested are summarized in Table 1.

**Table 1:** Treatments tested by synaptic calcium imaging.

Treatment	Description	Concentration	Application	Source
Phorbol 12,13-dibutyrate (PDBu)	Increases synaptic vesicle release via Munc13, PKC	1 μM	Acute	Sigma 524390
APV	NMDAR antagonist	50 μM	Acute	Tocris 0106
Glycine	NMDAR co-agonist	100 μM	Acute	Millipore 104201
NBQX	AMPAR antagonist	10 μM	Acute	Abcam ab120046
MGO-53	Control Antibody	15 μg/mL	Overnight	Harald Prüss (Wardemann et al., 2003)
NR1 003-102	Patient-derived NMDAR autoantibody	15 μg/mL	Overnight	Harald Prüss (Kreye et al., 2016)
NR1 007-124	Patient-derived NMDAR autoantibody	15 μg/mL	Overnight	Harald Prüss (Ly et al., 2018)
NR1 008-218	Patient-derived NMDAR autoantibody	15 μg/mL	Overnight	Harald Prüss (Ly et al., 2018)
Ketamine	NMDAR antagonist	1/10 μM	Acute [1 μM] Overnight [1/10 μM]	Sigma K2753
Memantine	Extrasynaptic NMDAR antagonist	1.5/10 μM	Acute [1.5 μM] Overnight [1.5/10 μM]	Sigma M9292
DMSO	Vehicle control	1:1,000	Acute/chronic	Supelco 102952

### ROI detection

Synaptic calcium transients were automatically detected using the calcium imaging analysis tool, Suite2p (Pachitariu et al., 2016). The input parameters for Suite2p were optimized for synapse detection by changing key parameters summarized in Table 2. ROIs detected by Suite2p were automatically filtered to remove noise by excluding those with a fluorescence skew—defined by Suite2p as the deviation of the ROI fluorescence from its neuropil—greater than 1. Only ROIs with a compactness less than or equal to 1.4 (where 1 is a perfect circle) were considered as synaptic calcium transients. More oblong dendritic events were detected but largely excluded from analysis since they were not limited to spines. Both skew and compactness values for each ROI were automatically generated by Suite2p and stored in ‘stat.npy’ files.

**Table 2:** Suite2p ROI detection parameter changes for synaptic calcium imaging.

Parameter category	Parameter	User input	Description
Functional detect	sparse_mode	1.0	Detect ROIs by searching for changes in fluorescence
	threshold_scaling	1.5	Threshold multiplier for ROI inclusion
	connected	1	Whether all pixels should be connected in the ROI
	max_overlap	0.9	Maximum amount of overlap allowed for ROIs in percent
	spatial_scale	0	Allows Suite2p to estimate the size of ROIs (for our data, spatial_scale was consistently estimated to be 6 pixels or ~2 μm spatial_scale = 1)
Extraction/neuropil	min_neuropil_pixels	50	Minimum number of pixels needed for neuropil fluorescence calculations
Classify/Deconv	spike_detect	0	Turn off deconvolution
Registration	do_registration	0	Stop image registration and motion correction. NOTE: If concatenating multiple videos of the same region, this must be enabled
Output settings	preclassify	0	Removes ROI exclusion by Suite2p classifiers; preserves all detected ROIs

### Signal extraction and normalization

A Python (Python Software Foundation, 2016) pipeline was created to automate preprocessing, Suite2p ROI detection, and analysis of fluorescence of each ROI. After potential ROIs are identified by Suite2p, the pipeline first converted the raw ROI fluorescence and neuropil fluorescence into a normalized change in fluorescence compared to baseline (ΔF/F_0_). To do this, 70% of the neuropil fluorescence was subtracted from the raw fluorescence of each ROI to correct for any potential neuropil contamination. To correct for bleaching and small fluorescence fluctuations, baseline correction was performed using an adaptive iteratively reweighted penalized least squares regression (airPLS; Zhang et al., 2010). The airPLS algorithm, used in spectral analyses such as NMR, iteratively measures the difference between its own baseline estimate and the original trace baseline. During the algorithm’s iterations, each point in the trace is reweighed based on the difference between these two values—peaks are given progressively lower weights, while baseline sections are given higher weights—resulting in a flat and corrected baseline. A conventional rolling median baseline correction can also be used (and is an option in the pipeline) but may not remove small fluctuations in the baseline and may be influenced by periods of high activity, resulting in negative baseline deflections.

### Baseline and calcium transient detection

Baseline fluorescence for normalization was determined using median absolute deviation (MAD; Leys et al., 2013). To calculate MAD, the absolute deviation of each data point in the fluorescence trace from the trace median is calculated as:

abs(trace point−trace median)

The median of these absolute deviations is then calculated to obtain an initial MAD value. Since the traces are primarily composed of noise, with a few distinct peaks occurring per ROI in each recording, the distribution of fluorescence values for any given calcium trace is right-skewed; however, the noise alone can be considered normally distributed.

The standard deviation is estimated by dividing the trace MAD by the constant 0.6745, specific to normally distributed data. This constant represents the relationship between MAD and the standard deviation (SD) for the middle 50% of a normal distribution, which corresponds to 0.6745 standard deviations. Therefore, the standard deviation (SD) can be calculated as:

$$\mathrm{SD}=\frac{\mathrm{MAD}}{0.6745}\;\text{or}\;\mathrm{SD}=\mathrm{MAD}\times 1.4826$$

This adjustment allows the standard deviation of a trace to be estimated from the MAD value. Frames with fluorescence values below MAD + 2*SD were considered to be noise. These data points without activity were used as a baseline reference (Mitani and Komiyama, 2018).

A refined MAD was then recalculated from the extracted baseline, without confounding peaks that were present in the original MAD calculation, to determine the baseline fluorescence (F_0_) and threshold for detecting peaks. A threshold of 4.5 standard deviations above F_0_ was set empirically based on manual examination of raw data, and used to detect peaks in each baseline-corrected, normalized trace using the `find peaks` function from the SciPy Python package (Virtanen et al., 2020). Constraints on peak detection were set: peaks could only occur greater than or equal to 100 ms (5 frames) apart, peak prominence, or deviation of a peak from local minima, was set to F_0_ + 2 × SD, and the peak half-width was set to be at least 3 frames. The ‘find peaks’ function with these constraints could accurately determine time stamps for when peaks occurred, allowing frequency and inter-event intervals to be calculated. The amplitude of each peak was calculated in relation to F_0_. ROIs with less than 2 detected transients were excluded from frequency analysis. All ROIs with at least one event were included in amplitude and synapse number analyses.

### Normalizing active synapses to neurite coverage

Cultures treated overnight with compounds and antibodies were compared to untreated control wells. To control for possible differences in neurite coverage, average projection images were first generated automatically using an ImageJ macro. Neurite coverage was measured automatically using CellProfiler v4.2.5 (Stirling et al., 2021). First, soma signals were masked and removed using the ‘Threshold’ and ‘MaskImage’ modules. The signal from neurites was enhanced using the ‘EnhanceOrSuppressFeatures’ and then thresholded and skeletonized using the ‘MorphologicalSkeleton’ module. The length of neurite coverage was calculated using ‘MeasureImageAreaOccupied’ and exported. The thresholded, skeletonized neurites were used to normalize synapse counts. Normalized synapses are reported per 10 μm of neurites.

### Statistical analysis

In 0 Mg^2+^ and TTX conditions, individual fields of view contained between 1,700 and 3,300 synapses; datasets contained between 150,000 and 340,000 individual synapses. To quantify the effect of pharmacological treatments on synaptic calcium transients, we employed generalized linear mixed-effect models (GLMMs) using R (R Core Team, 2025) and the glmmTMB package (Brooks et al., 2017). This was necessary due to the hierarchical nature of synaptic data. Imaged regions were highly heterogeneous and introduced variability; these were designated as random effects. Other potential random effects, such as culture preparation and well, were not included due to insufficient replicates for accurate modeling (*n* < 10). Datasets were always compared to relevant control conditions. Synaptic frequency values were analyzed using a generalized linear mixed-effects model fit with glmmTMB in R, where the treatment condition was considered the fixed effect and region as a random effect. Error was assumed to follow a Gamma distribution with a log link:

$$\log({\text{frequency}}_{\mathrm{ij}})=\beta_{0}+\beta_{1}{\text{Treatment}}_{i}+b_{j},$$

whereas average amplitude was also described using a GLMM, but by assuming a log-normal error distribution for average amplitude values:

$$\log\;({\text{amplitude}}_{\mathrm{ij}})=\beta_{0}+\beta_{1}{\text{Treatment}}_{i}+b_{j}.$$

where ${\text{frequency}}_{\mathrm{ij}}$ and ${\text{amplitude}}_{\mathrm{ij}}$ represent the expected frequency and average amplitude values, respectively, for synapse i in file j, $\beta_{0}$ is the intercept, $\beta_{1}$ represents the fixed effect of treatment condition, and $b_{j}$ is a random intercept accounting for the variability that exists between different regions imaged. Although mathematically similar, the two functions are distinct in assumptions of variance scaling: Gamma distributions assume that variance scales quadratically with the mean, whereas log-normal error distributions assume that the variance scales multiplicatively.

The accuracy of each model for each dataset was confirmed visually using R and DHARMa diagnostics (Hartig, 2024). Quantile-quantile plots were routinely examined to determine if the model accurately predicted simulated data values. Deviations of observed values from expected values, particularly for central values from 0.25 to 0.75 in quantile-quantile plots, indicated potential model misfit. Mixed-effects model results were compared to stratified, clustered bootstrap resampling with replacement, which was repeated 10,000 times. Bootstrap resampling consistently produced similar results to GLMM estimates and effect sizes.

Both mixed-effects models and bootstrap resampling results are visualized using forest plots, which show, on a log scale, the deviation of each treatment group from a reference group. Effect sizes are reported as a fold change relative to a reference group, with 1 being equivalent to the value of controls. Plots show the mean response for frequency or amplitude per group with 95% confidence intervals (CI). Results were considered statistically significant if the confidence interval did not cross one. In this way, true effects could be estimated in the event of model misfit. Wald *p*-values were generated for each model; however, they were not reported because they are influenced by model assumptions. Analysis and plot generations for mixed-effects models and bootstrap resampling were performed in R. Intraclass correlation coefficients (ICC) indicate the proportion of variance attributable to grouping synapses by imaged region. For each model, marginal and conditional R^2^ values are reported, representing the variance explained by fixed effects and by both fixed and random effects, respectively, together with the ICC.

To compare the number of active synapses before and after acute bath applications, a normality check was performed. For conditions with a singular control and acute treatment condition, if both groups of active synapses passed normality checks, a paired t-test was performed; if not, a Wilcoxon test was performed. Acute and chronic treatments with three or more groups were also checked for normality. Normally distributed synapse counts were analyzed with Friedman tests, while experiments that failed normality checks were analyzed with Kruskal-Wallis tests. Potential outliers were identified in synapse count data using the ROUT method (*Q* = 5%). Statistical analyses and plot generation for active synapses were done using GraphPad Prism 10 (version 10.5.0). Individual synaptic traces were visualized using matplotlib (Hunter, 2007), and raster plots were generated using pynapple (Viejo et al., 2023).

### Code availability

The code for synapse analysis in Python, neurite length measurement in CellProfiler, and R mixed-effect models is available here (https://github.com/jay-cee-begs/synaptic_suite2p).

## Results

**Figure 1:**
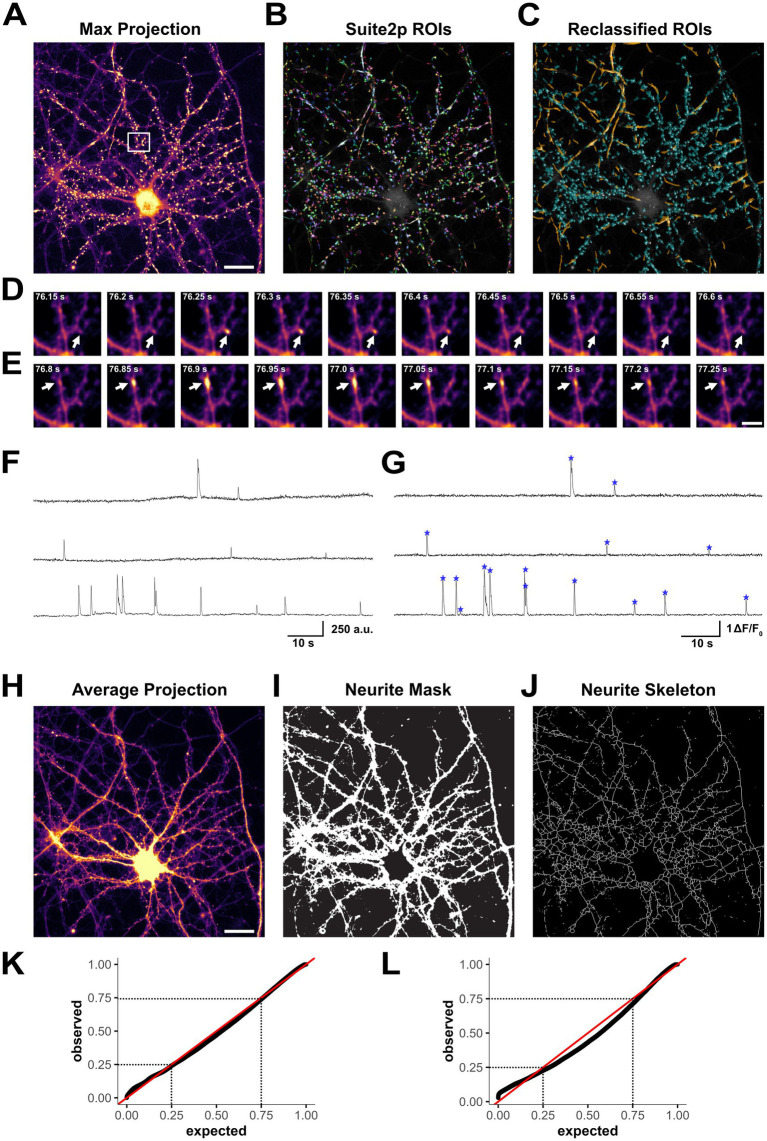
Synaptic calcium imaging analysis can be automated using Suite2p and Python. **(A)** Image of maximum fluorescence minus minimum fluorescence projection of a 3-min calcium imaging recording (scalebar = 25 μm). The region within the white box is expanded in panels **(D,E)**. **(B)** Image of maximum fluorescence generated by Suite2p overlayed with Suite2p-detected ROIs with random colors. **(C)** Reclassified synaptic (cyan) and dendritic (orange) spontaneous calcium transient ROIs. **(D)** Example time course of a representative synaptic calcium transient. **(E)** Example time course of a representative dendritic event (scalebar = 5 μm). **(F)** Three example raw fluorescence traces from three Suite2p-detected ROIs. **(G)** The same three example ROI traces shown in panel F, after airPLS baseline correction and normalization to ΔF/F_0_. Each detected peak is indicated by a blue asterisk. **(H)** Average fluorescence projection of a 3-min calcium imaging recording (scalebar = 25 μm). **(I)** Thresholded neurites and masked somatic signal generated by CellProfiler. **(J)** Skeletonized neurites generated by CellProfiler were used for normalizing synapse count per length neurite in chronically treated conditions. **(K)** Quantile-quantile plot of expected (black) and generated data points (red) for a well-fitting frequency mixed-effects model. **(L)** Quantile-quantile plot of expected (black) and generated data points (red) for a poorly-fitting frequency mixed-effects model.

**Figure 2:**
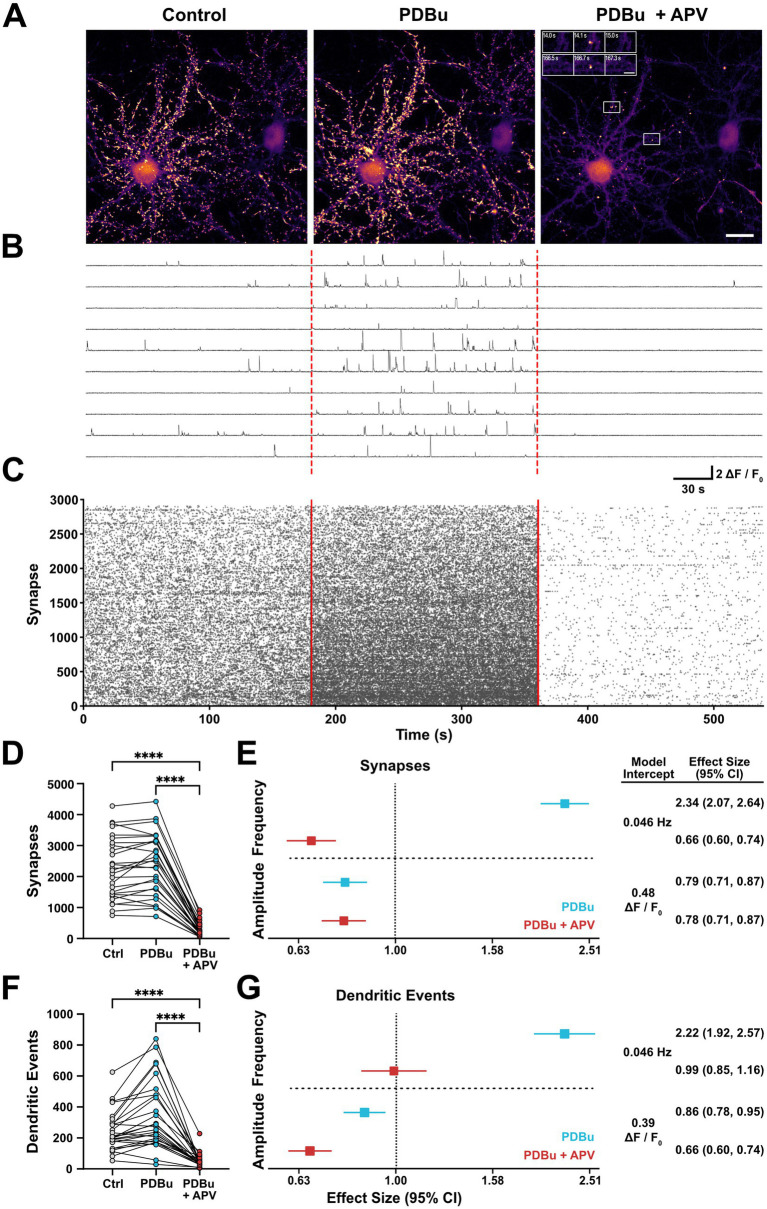
Phorbol esters increase, and APV decreases spontaneous synaptic calcium transient frequency. **(A)** Images of maximum fluorescence minus minimum fluorescence projections of 3 min calcium imaging recordings in control conditions (left), and in the presence of either 1 μM phorbol 12,13-dibutyrate (PDBu; middle) or 1 μM PDBu with 50 μM APV (right; scale bar = 25 μm). Two events at the top left for APV show that they are synaptic transients at synapses (scalebar = 5 µm). **(B)** Example concatenated ΔF/F_0_ traces from 10 selected synapses; red dashed lines indicate when each treatment began. **(C)** Raster plots showing activity across all synapses in baseline, PDBu, and PDBu plus APV treatment conditions; red solid lines indicate when each treatment began. **(D)** Comparisons of the number of spontaneously active synapses in each condition using a Friedman test (*n* = 57,872 control, 63,777 PDBu-treated, and 5,935 PDBu/APV-treated active synapses). **(E)** Forest plot results for synaptic event frequency (top) and amplitude (bottom) obtained from generalized linear mixed-effects models. PDBu (blue) and APV (red) are shown in reference to controls (vertical dashed line). Model intercepts indicate model-predicted averages for frequency or amplitude in control conditions. Effect sizes are reported as mean ± 95% confidence intervals (CI); raw values are summarized on the right. **(F)** Comparisons of the number of spontaneously active dendritic events in each condition using a Friedman test (*n* = 5,881 control, 9,096 PDBu-treated, and 833 PDBu/APV dendritic events). **(G)** Forest plot results for synaptic event frequency (top) and amplitude (bottom) obtained from generalized linear mixed-effects models. PDBu (blue) and APV (red) are shown in reference to controls (vertical dashed line). Model intercepts indicate model-predicted averages for frequency or amplitude in control conditions. Effect sizes are reported as mean ± 95% CI; raw values are summarized on the right. Model Metrics: Synapse frequency GLMM: ICC: 0.11, Marginal R^2^: 0.30, Conditional R^2^: 0.38. Synapse amplitude GLMM: ICC: 0.15, Marginal R^2^: 0.06, Conditional R^2^: 0.20. Dendritic event frequency GLMM: ICC: 0.12, Marginal R^2^: 0.22, Conditional R^2^: 0.32. Dendritic event amplitude GLMM: ICC: 0.13, Marginal R^2^: 0.05, Conditional R^2^: 0.17 (3 culture preparations; 9 wells imaged; 27 regions; DIV 19). *p < 0.05, **p < 0.01, ***p < 0.001, ****p < 0.0001.

To detect synaptic calcium transients, we transduced corticohippocampal neurons with synapsin promoter-driven GCaMP6f at DIV 0 and imaged between DIV 19 and 22 in 0 magnesium (Mg^2+^), to unblock NMDARs, and TTX to block action potentials. To automatically detect ROIs, we adapted Suite2p (Pachitariu et al., 2016) to detect spontaneous synaptic calcium transients en masse. Synaptic calcium transients were easily identifiable by their localized, large changes in fluorescence relative to baseline during live imaging (Supplementary Movie S1) and in maximum minus minimum projection images of live imaging over 3 min (Figure 1A). These spontaneous synaptic calcium transients can be automatically and reliably detected using Suite2p (Figure 1B) and can be further filtered to remove noise and longer dendritic events (Figure 1C). Dendritic events spread out from a singular point and last slightly longer than synaptic events, on average, whereas synaptic events most often appear limited to dendritic spines. Example time courses for a single synaptic (Figure 1D) and dendritic (Figure 1E) event are shown. Only the punctate, synaptic calcium transients were analyzed, except for a single experiment in which oblong, dendritic events were examined (Figure 2).

The raw fluorescence extracted from Suite2p for each ROI (Figure 1F) was then baseline corrected using an adaptive iteratively reweighted penalized least squares regression (airPLS; Zhang et al., 2010) and normalized (ΔF/F_0_) (Figure 1G). AirPLS correction shifted traces by an average of 0.044 ± 0.022 ΔF/F_0_, with baseline (0.044 ± 0.022 ΔF/F_0_) and peak periods (0.045 ± 0.025 ΔF/F_0_) affected similarly (calculated from 6 videos and 13,609 accepted ROIs). Calcium transients were detected using the scipy ‘find_peaks’ function and an empirically determined threshold of 4.5 standard deviations above F_0_ for peak detection (Figure 1G). Functional synapse number before and after treatment was quantified within the same imaged region in experiments with acute treatments. In experiments with overnight treatments, to account for potential differences in neuron/neurite coverage between randomly selected regions, we normalized functional synapse counts to neurite coverage, determined using GCaMP6f average projection images (Figure 1H) and CellProfiler pipelines. We removed somatic signal and created a binary mask of the neurites (Figure 1I). This mask was skeletonized (Figure 1J), and the number of synapses was normalized to the length of neurite coverage. Frequency and amplitude data were analyzed using mixed-effects models. Model accuracy was assessed using quantile-quantile plots that showed well-fitting (Figure 1K) or poorly-fitting (Figure 1L) mixed-effects models. All amplitude models were well-fitting whereas some frequency models were poorly-fitting. Results from poorly-fitting models were verified using bootstrap resampling.

To determine the rate of false negatives and false positives, we analyzed 12 randomly selected cropped areas (39 × 40 μm) from baseline recordings of 4 different imaged regions from each of 3 biological replicates. A total of 3,318 ROIs were detected where 2,912 (88.4 ± 4.5%) were accepted and 406 (15.6 ± 4.5%) were rejected. Of the 406 rejected ROIs, 321 were rejected because they failed to meet the skew (deviation of ROI fluorescence from surrounding fluorescence) threshold of ≥1, 11 because they failed to meet the peak criteria of at least 1 detected peak, and 74 for failing to meet both of these criteria. Inspection of rejected ROIs confirmed that 115 had plausible events, or a false negative rate of 3.7 ± 1.9%. All ROIs were detected by Suite2p, however, and were only rejected based on the criteria specified, which can be made more or less stringent by the user.

We found two false positives (ROIs automatically included that lacked plausible events) for a false positive rate of 0.3 ± 0.7%. We did not see any close proximity events falsely detected as a single event. Suite2p accounts for this by attempting to split each ROI into two ROIs to see if the variance in fluorescence can be explained more efficiently by two ROIs versus one ROI using an iterative k-means approach. Users have the ability to either allow or disallow overlapping pixels within ROIs. In our case, we disallowed overlap, meaning each ROI must recruit unique, neighboring pixels for it to be considered a separate ROI.

### Synaptic calcium transients increase in response to phorbol esters and are blocked by APV

To test if changes in functional synapse number and presynaptic release probability could be assayed using our automated analysis pipeline, we bath-applied the phorbol ester phorbol 12,13-dibutyrate (PDBu, 1 μM). PDBu interferes with Munc13 and protein kinase C (PKC) in the presynaptic terminal, which leads to an increase in presynaptic vesicle release probability (Lou et al., 2008). We then applied APV (50 μM) in sequence following phorbol ester application, to test whether synaptic transients are NMDA-dependent. APV is a preferential, potent, and well-established competitive antagonist of NMDARs (Collingridge et al., 1983).

Compared to control conditions, application of PDBu increased, and subsequent APV treatment decreased, synaptic fluorescence signal in maximum minus minimum projection images of all active synapses recorded during each treatment phase (Figure 2A; Supplementary Movie S2). Individual fluorescence traces showed changes in frequency across the three conditions at individual synapses (Figure 2B) and raster plots showed that these patterns persisted across all detected synapses (Figure 2C). PDBu did not change the number of active synapses compared to controls (~2,398 synapses; *p* = 0.40). APV addition decreased the number of active synapses approximately seven-fold (from ~2,268 synapses to ~319 synapses; *p* < 0.0001) compared to both control and PDBu-treated conditions (Figure 2D).

In control conditions, the average frequency was estimated to be 0.046 Hz. PDBu bath application acutely increased frequency to 0.11 Hz (2.34, 95% CI 2.07–2.64). APV decreased the frequency to 66% of control levels (0.031 Hz, 95% CI 0.60–0.74; Figure 2E, top). The average amplitude of synaptic calcium transients at baseline was 0.48 ΔF/F_0_ in control conditions. Average amplitude decreased in the presence of PDBu to 0.38 ΔF/F_0_, 79% of control (0.79, 95% CI 0.71–0.87). Subsequent addition of APV did not further decrease average amplitude compared to controls (0.78, 95% CI 0.71–0.87; Figure 2E, bottom). Together, these findings illustrate that synaptic calcium transients are dependent on presynaptic glutamate release and driven by NMDAR activity.

Dendritic calcium transients were also analyzed in control and PDBu and APV-treated conditions. Like synaptic events, dendritic calcium transients dramatically decreased in number in the presence of APV compared to both control and PDBu-treated conditions (*p* < 0.0001, Figure 2F). These events also responded to PDBu application with an increase in event frequency at individual synapses: at baseline, average dendritic event frequency for individual event sites was 0.046 Hz, and PDBu application increased frequency to 0.10 Hz (2.22, 95% CI 1.92–2.57). Interestingly, although APV application blocked ~82% of dendritic events, the remaining active sites had almost identical frequencies to baseline (0.99, 95% CI 0.85–1.16; Figure 2G, top). The amplitude of dendritic events was decreased in the presence of PDBu (0.86, 95% CI 0.78–0.95); however, dendritic event amplitude was then further decreased with the addition of APV (0.66, 95% CI 0.60–0.74), unlike synaptic events. Since dendritic events responded differently to APV than expected, they were not included in further analysis of NMDAR events, where we focused on synaptic events.

### Glycine increases synaptic calcium transient frequency and number

**Figure 3:**
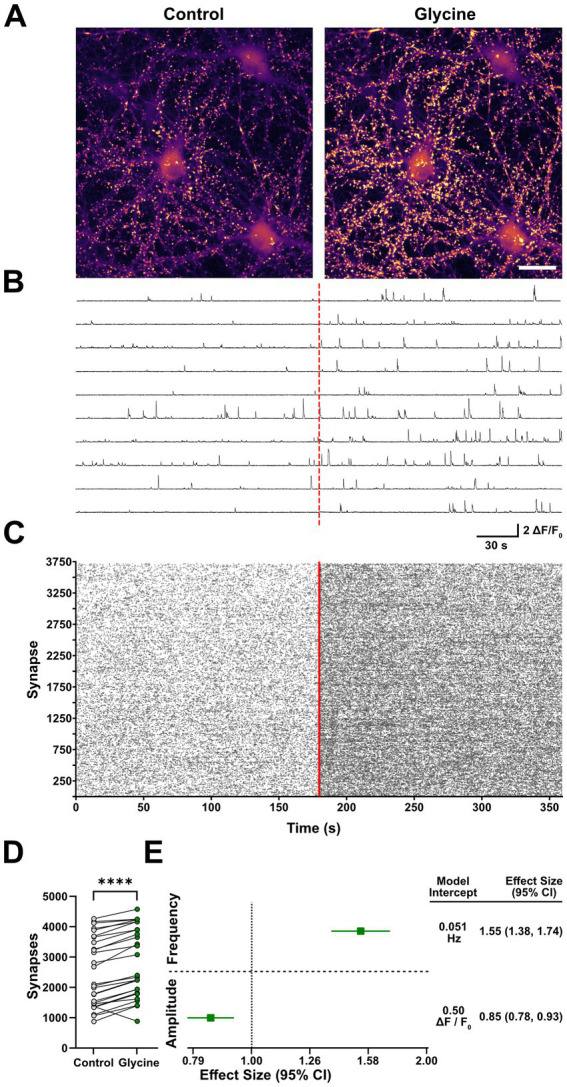
Glycine increases spontaneous synaptic calcium transient frequency and number of active synapses. **(A)** Images of maximum fluorescence minus minimum fluorescence projections of 3 min calcium imaging recordings in control conditions and in the presence of 100 μM glycine (right; scale bar = 25 μm). **(B)** Example concatenated ΔF/F_0_ traces from 10 selected synapses; the red dashed line indicates when treatment began. **(C)** Raster plots of activity across all synapses at baseline and with glycine treatment; the red solid line indicates when treatment began. **(D)** Comparisons of the number of spontaneously active synapses in each condition using a Wilcoxon Signed-Rank Test (*n* = 64,788 control, 75,509 glycine-treated active synapses). **(E)** Forest plot results for synaptic event frequency (top) and amplitude (bottom) obtained from generalized linear mixed-effects models. Glycine (green) is shown in reference to controls (vertical dashed line). Model intercepts indicate model-predicted averages for frequency or amplitude in control conditions. Effect sizes are reported as mean ± 95% CI; raw values are summarized on the right. Model Metrics: Frequency GLMM: ICC = 0.11, Marginal R^2^ = 0.11, Conditional R^2^ = 0.21. Amplitude GLMM: ICC = 0.12, Marginal R^2^ = 0.03, Conditional R^2^ = 0.14. (3 culture preparations; 9 wells; 27 regions; DIV 19). *p < 0.05, ** *p* < 0.01, *** *p* < 0.001, **** *p* < 0.0001.

To further test the NMDAR-dependence of synaptic calcium transients, we bath-applied the NMDAR co-agonist glycine at a concentration of 100 μM to cultures. This concentration is saturating for NMDARs (Cummings and Popescu, 2015). Glycine led to an immediate increase in fluorescence, which can be seen in maximum minus minimum projection images (Figure 3A). Traces from individual synapses (Figure 3B) and raster plots across all synapses (Figure 3C) revealed a dramatic increase in frequency of synaptic calcium transients in the presence of glycine. Glycine significantly increased the number of active synapses from ~2,503 to ~2,832 (*p* < 0.0001; Figure 3D). Furthermore, the frequency of synaptic calcium transient events increased by 55% from 0.051 Hz to 0.079 Hz (1.55, 95% CI 1.38–1.74; Figure 3E, top). The average amplitude of synaptic calcium transients simultaneously decreased by 15% from 0.50 to 0.43 ΔF/F_0_ (0.85, 95% CI 0.78–0.93; Figure 3E, bottom). The increase in number of active synapses and event frequency in response to glycine illustrates that synaptic calcium imaging reports multiple actions of NMDAR agonists, including postsynaptic changes in NMDA-receptor function and increases in functional synapse number.

### AMPA receptors have limited contribution to synaptic calcium transients

**Figure 4:**
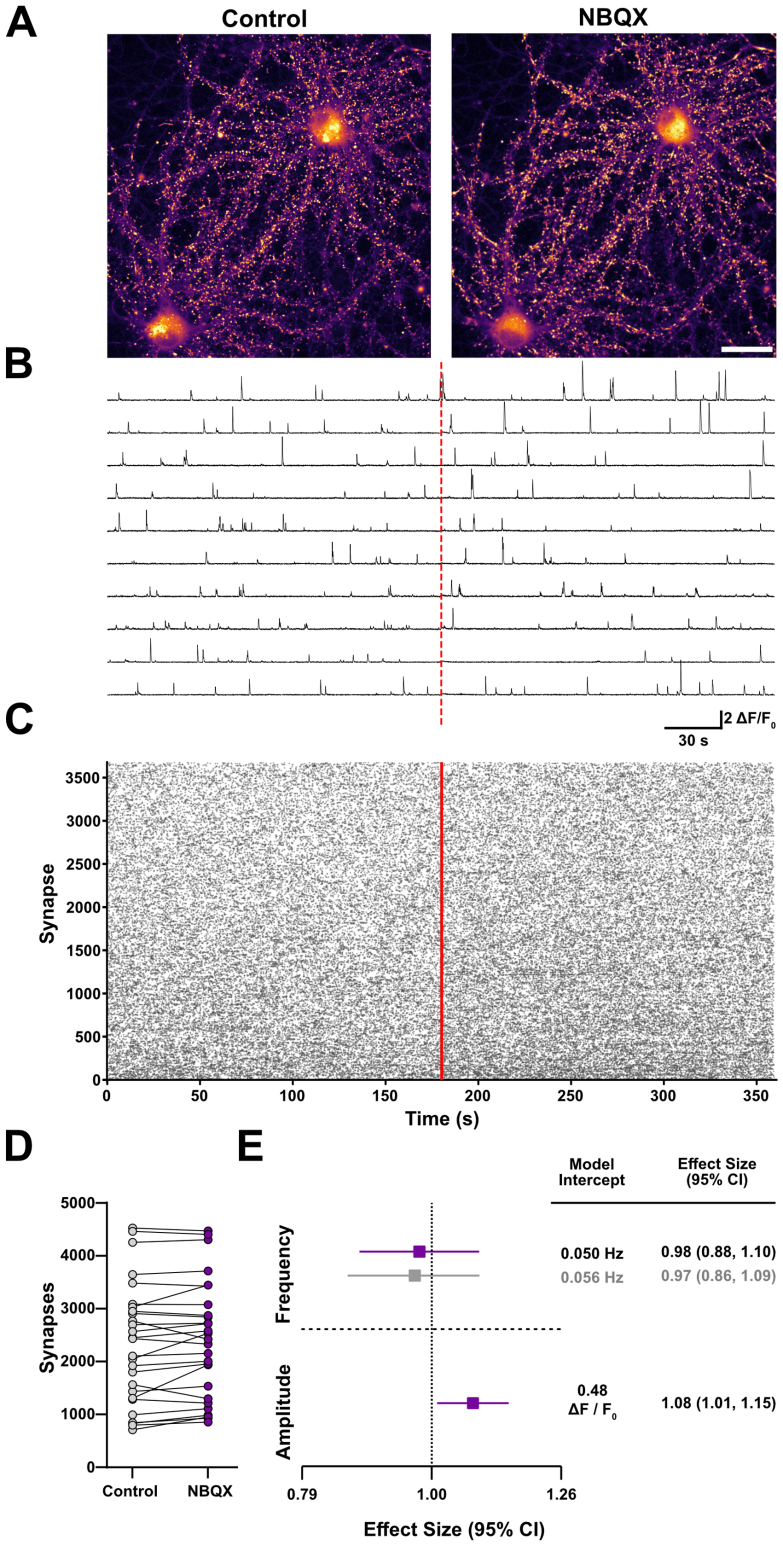
AMPARs show limited contribution to spontaneous synaptic calcium transients. **(A)** Images of maximum fluorescence minus minimum fluorescence projections of 3 min calcium imaging recordings in control conditions and in the presence of 10 μM NBQX (right; scale bar = 25 μm). **(B)** Example concatenated ΔF/F_0_ traces from 10 selected synapses; the red dashed line indicates when treatment began. **(C)** Raster plots of activity across all synapses at baseline and with glycine treatment. **(D)** Comparisons of the number of spontaneously active synapses in each condition using a paired *t*-test (*n* = 59,770 control, 61,988 NBQX-treated active synapses). **(E)** Forest plot results for synaptic event frequency (top) and amplitude (bottom) obtained from generalized linear mixed-effects models. NBQX (purple) is shown in reference to controls (vertical dashed line). The gray point in the frequency forest plot indicates the estimated mean ± 95% CI from bootstrap resampling (*n* = 10,000). Model intercepts indicate model-predicted averages for frequency or amplitude in control conditions from mixed-effects models (black) or bootstrap resampling (gray). Effect sizes are reported as mean ± 95% CI; raw values are summarized on the right for mixed-effects models (black) and bootstrap resampling (gray). Model Metrics: Frequency GLMM: ICC = 0.09, Marginal R^2^ = 0.00, Conditional R^2^ = 0.09. Amplitude GLMM: ICC = 0.06, Marginal R^2^ = 0.01, Conditional R^2^ = 0.06. (3 culture preparations; 9 wells imaged; 27 regions; DIV 19). *p < 0.05, **p < 0.01, ***p < 0.001, ****p < 0.0001.

To test if AMPA receptors (AMPARs) contribute to synaptic calcium transients, we bath-applied the AMPAR antagonist NBQX at a concentration of 10 μM. A general increase in fluorescence of synaptic events appeared following NBQX treatment (Figure 4A). An increase is also seen in the amplitude of individual events in single synaptic traces (Figure 4B), but no difference in event frequency is apparent in raster plots (Figure 4C). Comparison of synapse number between control and NBQX-treated conditions showed no significant difference, although there was a trend toward an increase in synapse number from ~2,331 to ~2,401 within each imaged region before and after treatment (Figure 4D, *p* = 0.088).

NBQX application had no significant effect on the frequency of synaptic events (0.98, 95% CI 0.88–1.10; Figure 4E, top). Bootstrap resampling supported GLMM results, verifying that NBQX application did not alter the frequency of synaptic calcium transients (Figure 4E, top; gray). Inhibition of AMPARs did, however, significantly increase the average amplitude of synaptic calcium transients from 0.48 ΔF/F_0_ to 0.52 ΔF/F_0_ (1.08, 95% CI 1.01–1.15).

### Acute effects of NMDAR-targeting compounds: ketamine and memantine reduce synaptic activity

After demonstrating that synaptic calcium imaging can report increases and decreases in numbers of functional synapses and changes in presynaptic release, in addition to postsynaptic effects, we next sought to test the potential of this pipeline for screening the NMDAR-targeting compounds ketamine and memantine. These compounds were tested for acute and overnight effects. The IC_50_ for ketamine and memantine were measured in rat hippocampal neuronal culture using electrophysiology to be 0.43 ± 0.10 μM and 1.04 ± 0.26 μM, respectively (Parsons et al., 1996). We initially decided to test concentrations slightly higher than this: 1 μM for ketamine and 1.5 μM for memantine.

**Figure 5:**
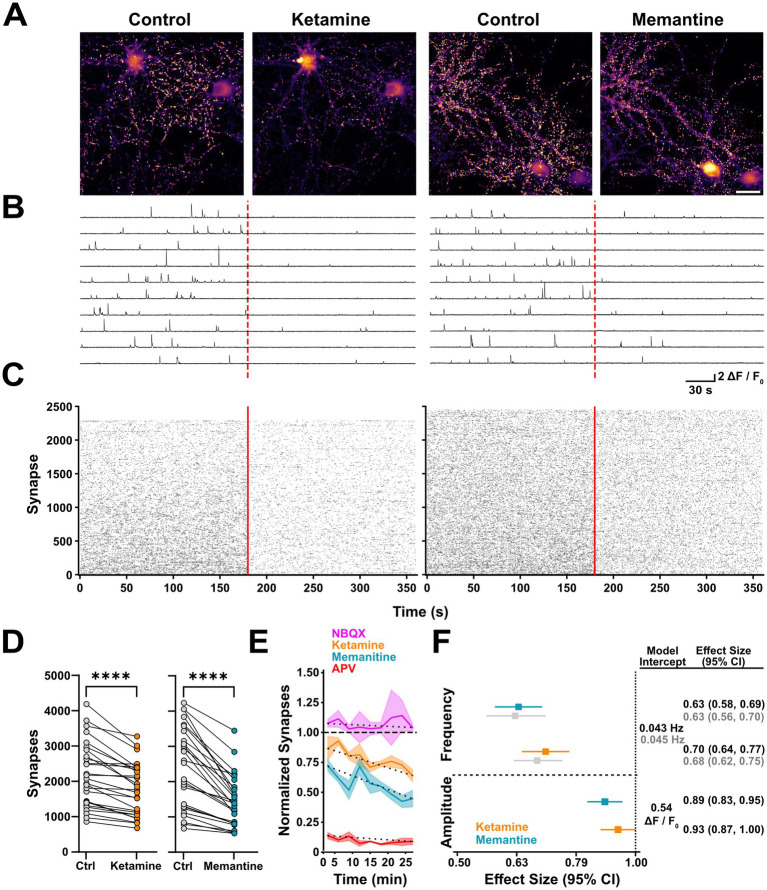
Ketamine and memantine decrease spontaneous synaptic calcium transient frequency and amplitude. **(A)** Images of maximum fluorescence minus minimum fluorescence projections of 3 min calcium imaging recordings in control conditions and in the presence of either 1 μM ketamine (left) or 1.5 μM memantine (right; scale bar = 25 μm). **(B)** Example concatenated ΔF/F_0_ traces from 10 randomly selected synapses; the red dashed lines indicate when each treatment began. **(C)** Raster plots of activity across all synapses at baseline and with either ketamine (left) or memantine (right) treatment; the red solid lines indicate when each treatment began. **(D)** Comparisons of the total number of spontaneously active synapses in control (*n* = 55,397) and ketamine conditions (*n* = 42,691) using a paired *t*-test (left) and active synapses in control (*n* = 61,143) and memantine (*n* = 32,548) conditions using a Wilcoxon signed-rank test (right). **(E)** Line plots showing the proportion of active synapses after treatment, normalized to active synapses at baseline in the same region. **(F)** Forest plot results for synaptic event frequency (top) and amplitude (bottom) obtained from generalized linear mixed-effects models. Ketamine (orange) and memantine (cyan) are shown in reference to DMSO vehicle controls (vertical dashed line). Bootstrap results for each treatment group frequency (gray) are shown directly below the mixed-effects model results. Model intercepts indicate model-predicted averages for frequency or amplitude in control conditions from mixed-effects models (black) or bootstrap resampling (gray). Effect sizes are reported as mean ± 95% CI; raw values are summarized on the right for mixed-effects models (black) and bootstrap resampling (gray). Model metrics: Frequency GLMM: ICC = 0.08, Marginal R^2^ = 0.10, Conditional R^2^ = 0.17. Amplitude GLMM: ICC = 0.06, Marginal R^2^ = 0.01, Conditional R^2^ = 0.07. (3 culture preparations; 9 wells imaged; 27 regions; DIV 19). *p < 0.05, **p < 0.01, ***p < 0.001, ****p < 0.0001.

Ketamine or memantine was bath applied to cultures in 0 mM Mg^2+^ and TTX acutely. In maximum minus minimum fluorescence projection images, a marked decrease in the number of active synapses was observed for both compounds (Figure 5A). Fluorescence traces of individual synapses also showed a decrease in the frequency and amplitude of synaptic calcium transients after treatment compared to baseline (Figure 5B). In raster plots we also observed a decrease in the density of synaptic calcium transients in treated conditions compared to control conditions across all synapses (Figure 5C). Both ketamine and memantine decreased the total number of active synapses. Ketamine decreased the number of active synapses from ~2,179 at baseline to ~1,769 after treatment (*p* < 0.0001; Figure 5D, left) while memantine decreased the number of active synapses from ~2,376 to ~1,427 (*p* < 0.0001; Figure 5D, right). Acute application of DMSO vehicle did not significantly change the number of active synapses (baseline: ~1,800, treated: ~1,868, *p* = 0.40; data not shown).

Imaging multiple regions in sequence enabled us to track ketamine and memantine antagonism of NMDARs over time. Percentages of active synapses after treatment were normalized to the number of active synapses in the same region at baseline. Each image, in sequence, allowed us to track how the proportion of active synapses changed over time after acute bath applications. We observed that ketamine and memantine progressively increase effects on synapses over time, whereas APV and NBQX produce immediate and stable effects over 30 min. (Figure 5E). Following ketamine treatment, 88.8 ± 17.8% of synapses were active in the first 2–3 min after application, and 63.9 ± 9.0% of synapses were active in the last imaged region 30 min later (orange). In the first 2–3 min after memantine application, the proportion of active synapses compared to baseline was 72.4 ± 8.7%, and in the last region imaged, the proportion dropped to 44.7 ± 12.1%. The decrease in active synapses over time is supported by previous evidence that both ketamine and memantine antagonism of NMDARs only occurs after channel opening (Chen et al., 1992; MacDonald et al., 1987). Memantine is also known to have faster blocking kinetics than ketamine (Parsons et al., 1995), although ketamine has a higher affinity for NMDARs. APV antagonism has faster effects, with only 13.8 ± 4.7% active synapses remaining immediately after acute application and 9 ± 4.8% active synapses approximately 30 min later. Conversely, NBQX addition increased synapse number immediately to 108 ± 5.4%, and this increase was maintained until the last imaging session, with 104 ± 5.2% of baseline synapses after approximately 30 min. Rather than imaging a single region for 30 min to track inhibition of individual synapses over time, in multiple regions imaged in sequence we can detect a decrease in the number of active synapses over time, after acute treatment of ketamine or memantine, relative to a baseline reference.

Compared to controls, both ketamine and memantine decreased synaptic calcium transient frequency. The frequency in DMSO vehicle-treated control conditions was 0.043 Hz. Ketamine decreased frequency to 0.030 Hz (0.70, 95% CI 0.64–0.77) and memantine decreased frequency to 0.027 Hz (0.63, 95% CI 0.58–0.69) (Figure 5F, top). Bootstrap resampling supported frequency model results with similar effects observed in each (Figure 5F, top; gray). The average amplitude of synaptic calcium transients in DMSO controls was 0.54 ΔF/F_0_. Acute ketamine treatment trended toward decreased average amplitude (0.93, 95% CI 0.87–1.00) while memantine decreased average amplitude to 0.48 ΔF/F_0_ (0.89, 95% CI 0.83–0.95; Figure 5F, bottom). DMSO vehicle did not change the frequency or amplitude of synaptic events acutely (data not shown).

### Chronic ketamine treatment reduces the frequency and amplitude of synaptic calcium transients

**Figure 6:**
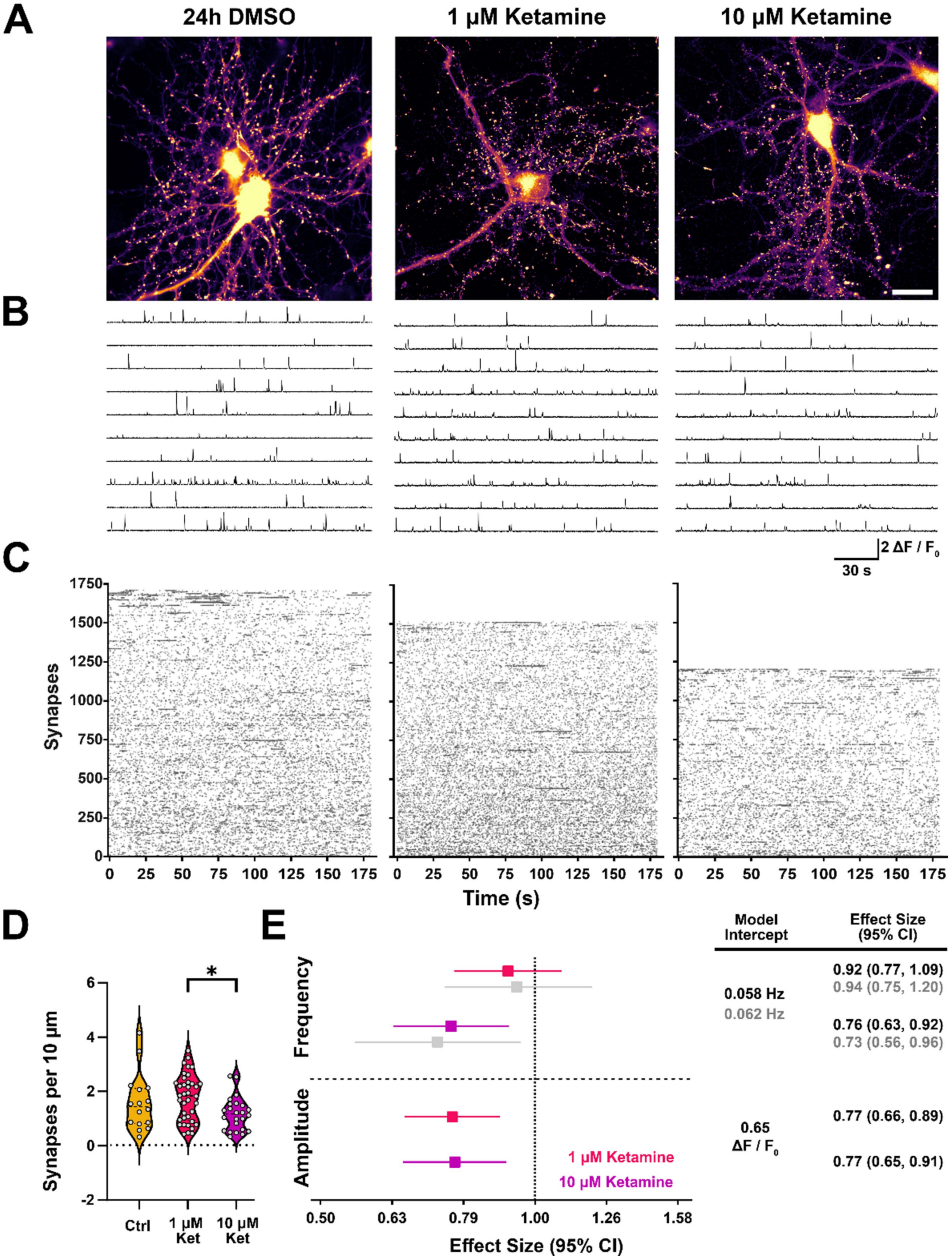
Overnight ketamine treatment reduces the frequency and amplitude of synaptic events. **(A)** Images of maximum fluorescence minus minimum fluorescence projections of 3 min synaptic calcium imaging recordings of regions treated overnight with a DMSO vehicle (left), 1 μm ketamine (middle), or 10 μm ketamine (right; scale bar = 25 μm). **(B)** Example ΔF/F_0_ traces from 10 randomly selected synapses from each condition. **(C)** Raster plots of activity across all synapses in each treatment condition. **(D)** Comparisons of the number of spontaneously active synapses in each condition per 10 μm of GCaMP6f neurite coverage using a Kruskal-Wallis test. **(E)** Forest plot results for synaptic event frequency (top) and amplitude (bottom) obtained from generalized linear mixed-effects models. 1 μm ketamine (red) and 10 μm ketamine (purple) are shown in reference to 24 h DMSO vehicle controls (vertical dashed line). Bootstrap results for each ketamine treatment for frequency (gray) are shown directly below mixed-effects model results. Model intercepts indicate model-predicted averages for frequency or amplitude in control conditions from mixed-effects models (black) or bootstrap resampling (gray). Effect sizes are reported as mean ± 95% CI, raw values are summarized on the right for mixed-effects models (black) and bootstrap resampling (gray). Model Metrics: Frequency GLMM: ICC = 0.12, Marginal R^2^ = 0.03, Conditional R^2^ = 0.14. Amplitude GLMM: ICC = 0.19, Marginal R^2^ = 0.02, Conditional R^2^ = 0.20. (35 culture preparations; 15–38 regions; DIV 19 and DIV 22). *p < 0.05, **p < 0.01, ***p < 0.001, ****p < 0.0001.

Since ketamine is tested clinically for its antidepressant actions, we also tested what synaptic changes could occur with overnight exposures of cultures to ketamine. Ketamine was applied overnight at 1 μM and an order of magnitude more, at 10 μM. Cultures remained in ketamine until they were switched into a 0 Mg^2+^ TTX-containing solution without ketamine for synaptic calcium imaging. Maximum minus minimum projection images (Figure 6A) and synaptic transient fluorescence traces (Figure 6B) showed a concentration-dependent decrease in synaptic calcium transient amplitude in synapses treated overnight with ketamine. Raster plots of activity across all detected synapses hint at both a decreased density of events and fewer synapses in regions treated with 10 μM ketamine overnight compared to control and 1 μM ketamine treatment (Figure 6C). Interestingly, overnight ketamine treatment did not significantly change synapse number per 10 μm of neurites compared to DMSO vehicle controls (1 μM ketamine: *p* = 0.91, 10 μM ketamine: *p* = 0.69). However, there was a concentration-dependent effect of overnight ketamine treatment, with 10 μM ketamine leading to less active synapses compared to 1 μM ketamine (*p* = 0.023, Figure 6D).

Overnight treatment with DMSO vehicle produced an average frequency of 0.058 Hz. Only 10 μM ketamine significantly decreased calcium transient frequency to 0.044 Hz (0.76, 95% CI 0.63–0.92; Figure 6E top). Mixed-effects results were compared to bootstrap resampling, which showed similar effects (Figure 6E top, gray). The amplitude of synaptic calcium transients was also compared between DMSO and the two concentrations of ketamine. Interestingly, both 1 μM and 10 μM ketamine decreased synaptic calcium transient amplitude equally from 0.65 ΔF/F_0_ to 0.50 ΔF/F_0_ (1 μM ketamine: 0.77, 95% CI 0.66–0.89; 10 μM ketamine: 0.77, 95% CI 0.65–0.91; Figure 6E, bottom).

### Chronic memantine treatment reduces synaptic calcium transient amplitude

**Figure 7:**
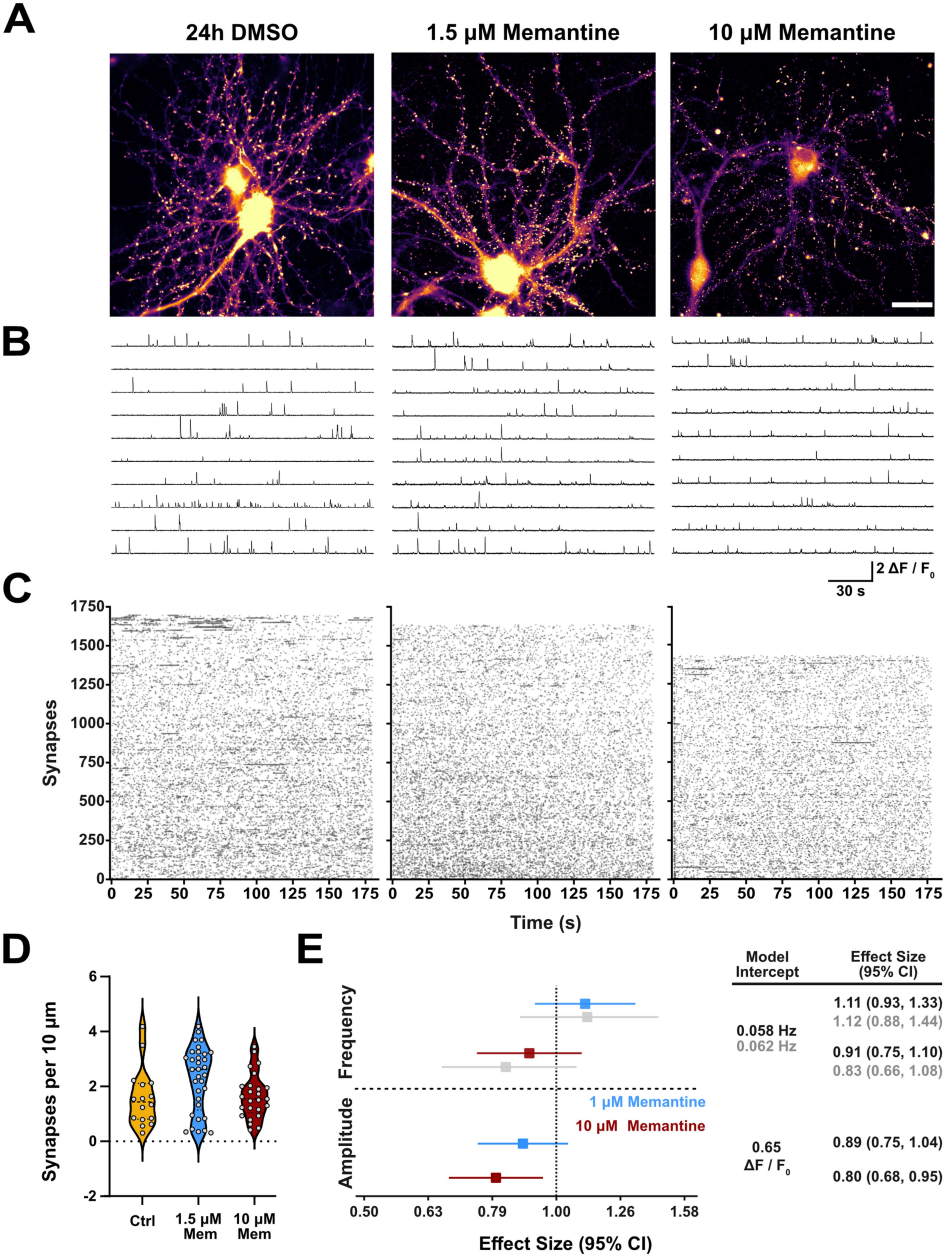
Overnight memantine treatment decreases the amplitude of synaptic events. **(A)** Images of maximum fluorescence minus minimum fluorescence projections of 3 min synaptic calcium imaging recordings of regions treated overnight with a DMSO vehicle (left), 1.5 μM memantine (middle), or 10 μM memantine (right; scale bar = 25 μm). **(B)** Example ΔF/F_0_ traces from 10 randomly selected synapses from each condition. **(C)** Raster plots of activity across all synapses in each treatment condition. **(D)** Comparisons of the number of spontaneously active synapses in each condition per 10 μm of GCaMP6f neurite coverage using a Kruskal-Wallis test. **(E)** Forest plot results for synaptic event frequency (top) and amplitude (bottom) obtained from generalized linear mixed-effects models. 1.5 μM memantine (blue) and 10 μM memantine (burgundy) are shown in reference to 24 h DMSO vehicle controls (vertical dashed line). Bootstrap results for each memantine treatment for frequency (gray) are shown directly below mixed-effects model results. Model intercepts indicate model-predicted averages for frequency or amplitude in control conditions from mixed-effects models (black) or bootstrap resampling (gray). Effect sizes are reported as mean ± 95% CI, raw values are summarized on the right for mixed-effects models (black) and bootstrap resampling (gray). Model Metrics: Frequency GLMM: ICC = 0.12, Marginal R^2^ = 0.03, Conditional R^2^ = 0.14. Amplitude GLMM: ICC = 0.19, Marginal R^2^ = 0.02, Conditional R^2^ = 0.20.(35 culture preparations; 15–29 regions; DIV 19 and DIV 22). *p < 0.05, **p < 0.01, ***p < 0.001, ****p < 0.0001.

**Figure 8:**
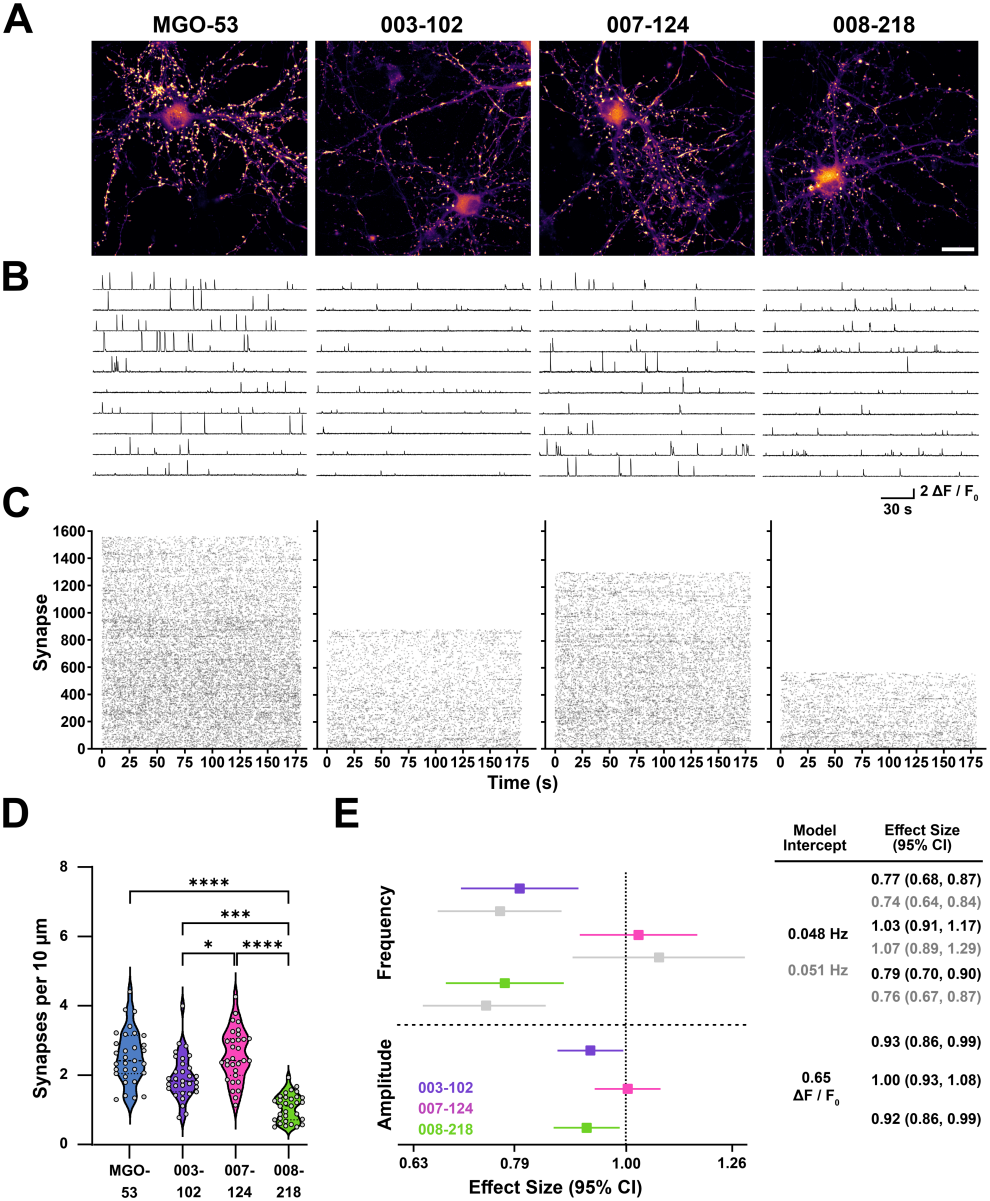
Encephalitis patient-derived anti-NMDAR autoantibodies reduce the number of active synapses and impair synaptic calcium transient frequency and amplitude. **(A)** Images of maximum fluorescence minus minimum fluorescence projections of 3 min synaptic calcium imaging recordings of regions treated overnight with 15 μg/ml of either control antibody (MGO-53, left), or patient-derived NMDAR autoantibodies (003-102, 007-124, and 008-218; right; scale bar = 25 μm). **(B)** Example ΔF/F_0_ traces from 10 selected synapses from each condition. **(C)** Raster plots of activity across all synapses in each treatment condition. **(D)** Comparisons of the number of spontaneously active synapses in each condition per 10 μm of GCaMP6f neurite coverage using the non-parametric Kruskal-Wallis test. **(E)** Forest plot results for synaptic event frequency (top) and amplitude (bottom) obtained from generalized linear mixed-effects models. Antibody treatments 003-102 (purple), 007-124 (pink), and 008-218 (green) are shown in reference to MGO 53 controls (vertical dashed line). Bootstrap results for each treatment group frequency are shown in gray directly below the mixed-effects model results. Model intercepts indicate model-predicted averages for frequency or amplitude in control conditions from mixed-effects models (black) or bootstrap resampling (gray). Effect sizes are reported as mean ± 95% CI; raw values are summarized on the right for mixed-effects models (black) and bootstrap resampling (gray). Model Metrics: Frequency GLMM: ICC = 0.12, Marginal R^2^ = 0.03, Conditional R^2^ = 0.14. Amplitude GLMM: ICC = 0.06, Marginal R^2^ = 0.005, Conditional R^2^ = 0.06. (4 culture preparations; 15 wells imaged; 31 regions; DIV 20). *p < 0.05, **p < 0.01, ***p < 0.001, ****p < 0.0001.

Like ketamine, we also tested overnight treatment of memantine at 1.5 μM and 10 μM. Maximum minus minimum projection images showed a decrease in fluorescence of synaptic calcium transients in regions treated with 10 μM memantine compared to controls and 1.5 μM memantine (Figure 7A). Traces from individual synapses suggested a concentration-dependent decrease in synaptic calcium transient amplitude with increasing concentrations of memantine (Figure 7B). Raster plots of total activity did not reveal any obvious changes in frequency of events (Figure 7C). Overnight memantine treatment did not change the number of active synapses (Figure 7D), or synaptic calcium transient frequency, at either concentration; 1.5 μM memantine trended toward increasing frequency (1.11, 95% CI 0.93–1.33) while 10 μM trended toward decreasing frequency (0.91, 95% CI 0.75–1.10) (Figure 7E, top). Bootstrap resampling supported model results of no significant difference in frequency, and the trend in difference in effect direction with 1.5 μM and 10 μM memantine (Figure 7E, top; gray). Treatment with 10 μM memantine decreased the amplitude of calcium transients compared to controls (0.80, 95% CI 0.68–0.95; Figure 8E, bottom). The effect of memantine on synaptic calcium transients is weaker overnight compared to acute applications in 0 Mg^2+^. This is likely because memantine binds NMDARs more weakly in the presence of 1 mM Mg^2+^ (Kotermanski and Johnson, 2009), which was present during overnight treatment in Neurobasal-A medium.

### Synaptic calcium imaging detects differential effects of patient-derived NMDAR autoantibodies

Lastly, we investigated three encephalitis patient-derived anti-NMDAR autoantibodies to test whether our pipeline could detect differences in effects on synapses. The antibodies tested were 003-102, 007-124, and 008-218 (Kreye et al., 2016; Ly et al., 2018) compared to MGO-53 as a negative control (Kreye et al., 2016; Michalski et al., 2024; Wardemann et al., 2003). MGO-53 is a non-reactive patient antibody from mature, naïve B-cells (Wardemann et al., 2003) and was chosen as a control to compare with previous studies using these same patient autoantibodies (Kreye et al., 2016; Michalski et al., 2024).

Cultures were treated overnight with patient antibodies at a concentration of 15 μg/mL, as in Kreye et al. (2016), and then imaged in 0 mM Mg^2+^ ACSF with TTX. In maximum minus minimum projection images (Figure 8A) and in synaptic calcium traces (Figure 8B), two antibodies, 003-102 and 008-218, decreased synaptic calcium transients compared to MGO-53 negative controls. The frequency of activity in cultures treated with antibodies 003-102 and 008-218 and number of active synapses also appeared decreased compared to MGO-53 controls and antibody 007-124, in raster plots (Figure 8C). Antibody 007-124 did not change the number of active synapses compared to MGO-53 controls (*p* > 0.99; Figure 8D), in line with its lower affinity compared to 003-102 and 008-218 (Ly et al., 2018). The number of active synapses trended toward a decrease after treatment with antibody 003–102 (*p* = 0.066; exclusion of one outlier resulted in *p* = 0.034) and was significantly decreased by antibody 008-218 (*p* < 0.0001). The finding that antibody 008-218 decreases the number of active synapses more than 003-102 is surprising, given that antibody 003-102 has a similar binding affinity for NMDARs compared to antibody 008-218 (Ly et al., 2018).

Patient antibodies 003-102 and 008-218 decreased the frequency of synaptic calcium transients at individual synapses compared to MGO-53 control treatments, while antibody 007-124 did not (Figure 8E, top). The frequency of MGO-53-treated controls was 0.048 Hz. Antibody 003-102 decreased frequency to 0.039 Hz, by 21% compared to controls (0.79, 95% CI 0.70–0.90), and antibody 008–218 decreased frequency to 0.037 Hz, by 23% (0.77, 95% CI 0.68–0.87). Bootstrap resampling confirmed mixed-effects model results: both 003-102 and 008-218 antibodies decreased the frequency of synaptic calcium transients compared to MGO-53, while 007-124 showed no change (Figure 8E, top; gray). The amplitude of synaptic calcium transients in control conditions was estimated to be 0.65 ΔF/F_0_. Antibodies 003-102 and 008-218 both decreased amplitude to 0.60 ΔF/F_0_ (003-102: 0.93, 95% CI 0.86–0.99; 008-218: 0.92, 95% CI 0.86–0.99). Antibody 007-124 did not affect amplitude (similar to its lack of effect on active synapse number and frequency, which were also unchanged).

### Potential use of synaptic calcium imaging as a high-throughput screen

For our pipeline to be usable as a high-throughput screening platform, reproducibility and ability to detect effects are important, usually reported as a Z’ (Z-prime) score. We first examined the sources of variability in the pipeline by calculating the standard deviation of frequency, amplitude, and active synapse number (in baseline conditions) for synapses, imaged regions, wells, and replicates (Table 3). Synapses were the most variable, as reported in previous studies (Walker et al., 2017; Metzbower et al., 2019), while replicates were the least variable for frequency and amplitude. Active synapse number was similarly variable across imaged regions, wells, and replicates.

**Table 3:** Variability of parameters at synapse, image, well, and replicate levels.

Standard deviation	Frequency	Amplitude	Active synapse number
Synapses (399,575)	0.0486 ± 0.0458	0.560 ± 0.329	Not applicable
Imaged regions (162)	0.0464 ± 0.0126	0.563 ± 0.090	2,243.04 ± 1,055.56
Wells (54)	0.0469 ± 0.0101	0.563 ± 0.064	2,334.04 ± 959.79
Replicates (3)	0.0482 ± 0.0021	0.561 ± 0.016	2,412.49 ± 1,040.81

We then calculated Z’ using mean/standard deviation, and using median/MAD, which is insensitive to outliers, since event distributions were non-Gaussian, for frequency, amplitude, active synapse number (per length dendrite), and integrated fluorescence (which combines active synapses, frequency, and amplitude), using positive (max signal) and negative (min signal) controls for each parameter (Memantine/PDBu for frequency and events, APV/NBQX for amplitude, APV/glycine for active synapse number, and APV/PDBu for integrated fluorescence). Z’ values were calculated by grouping synapses by image, well, or replicate (Table 4).

**Table 4:** Z’ for different parameters across imaged regions, wells and replicates.

Z’	Parameter	Image (27)	Well (9)	Replicate (3)
Mean/SD	Frequency	−0.006	0.212	0.672
	Amplitude	−2.626	−2.199	−1.584
	Active synapses	−0.130	0.307	0.543
	Integrated fluorescence	−0.642	−0.088	0.296
Median/MAD	Frequency	−0.263	0.252	0.874
	Amplitude	−3.294	−1.855	−0.496
	Active synapses	−0.254	0.409	0.592
	Integrated fluorescence	−0.976	0.313	0.275

Our assay had a Z’ between 0 and 0.5 for frequency/well, active synapses/well and integrated fluorescence/well and replicate, and a Z’ > 0.5 for frequency/replicate and active synapses/replicate. These values are within a range that is usable for a cell-based high-throughput screen.

## Discussion

Our automated calcium imaging ROI detection and analysis pipeline reports multiple parameters of synapse function, including the number of functional synapses and pre and postsynaptic function, for thousands of individual synapses. Using this pipeline, we collected data from 1,198,748 spontaneously active synapses and detected 10,228,773 unique synaptic calcium transient events without any manual ROI selection. We were able to attain such a dataset due to optimized automated analysis, in addition to semi-automated imaging, allowing multiple regions to be imaged in sequence on glass-bottom 24-well plates, which could be extended to 96- and 384-well plate formats. To accommodate such large datasets, we analyzed data using generalized linear mixed-effects models. These models were consistently comparable to stratified, clustered bootstrap resampling with replacement, indicating that both statistical tests give comparable results when analyzing average amplitude and frequency of synaptic calcium transient events. Examination of mixed-effects models supported modeling of the imaged region as a random effect; however, most of the variability in the data existed between synapses within the same field of view, rather than between regions.

Our synaptic calcium imaging analysis pipeline also has potential as a high-throughput screen. Our assay had a Z’ between 0 and 0.5 for frequency/well, active synapses/well and integrated fluorescence/well and replicate, and a Z’ > 0.5 for frequency/replicate and active synapses/replicate. A good Z’ for a cell-based assay (which is inherently more variable than biochemical assays) is between 0 and 0.5 (An and Tolliday, 2010; Bar and Zweifach, 2020) and values above 0.5 are excellent. Thus, our pipeline is usable as a high-throughput screen. Nevertheless, it would likely serve as a secondary medium-throughput readout given the time it takes to perform live imaging across multiple wells and/or regions and replicates.

We tested imaging of synaptic calcium transients using bath-applied dyes, i.e., Calbryte 590, Calbryte 520, Cal-520, and OGB-1. However, the number of detected synapses was much lower, likely in part because dye in astrocytes increases background signal, and signal bleached much more compared to GCaMP6f samples. It is possible that with optimization, synaptic calcium transients could be detected and quantified using improved calcium dyes. Currently, we advise using GECIs for synaptic calcium imaging (e.g., GCaMP6 or GCaMP3 used here and in all other publications using this method).

We found that compounds that modulate postsynaptic receptors can also alter synaptic calcium transient frequency, but they simultaneously alter synapse number. For example, acute addition of glycine, a co-agonist of NMDARs, increased both frequency and synapse number, while acute addition of NMDAR antagonists such as ketamine, memantine, or APV decreased both frequency and number of active synapses. To differentiate whether changes in frequency are due to presynaptic or postsynaptic changes, reporting total active synapse numbers is crucial. All direct NMDAR agonists and antagonists changed the number of active synapses drastically within the same field of view compared to baseline recordings (*p* < 0.0001), whereas presynaptic modulators such as PDBu did not change the number of active synapses.

The blockade of synaptic calcium transients in the presence of APV verifies that they are NMDAR-dependent and aligns with results from previous studies (Andreae and Burrone, 2015; Metzbower et al., 2019; Prikhodko et al., 2024; Sinnen et al., 2016; Walker et al., 2017). APV eliminated 92% of events compared to control, and 97% of events compared to PDBu treatment, in our experiments, similar to 94% of events eliminated by APV in Metzbower et al. (2019). The involvement of AMPARs in synaptic calcium transients is less clear. The AMPAR antagonist NBQX did not significantly change the frequency of calcium transients at individual synapses. However, there was a trend toward an increase in the number of active synapses (*p* = 0.088). Surprisingly, we observed an increase in amplitude of synaptic calcium transients in the presence of NBQX, contradicting results from previous studies (Walker et al., 2017; Metzbower et al., 2019, mentioned in text but data not shown). The observed increase in calcium transient amplitude could be attributed to the larger sample size investigated here and the use of automated ROI detection rather than manual detection. Biologically, the absence of changes in synapse number or event frequency in the presence of NBQX suggests that AMPARs do not flux calcium directly, but instead modulate calcium amplitude via NMDAR-AMPAR cross-talk. Using electrophysiology in hippocampal neurons, Bai et al. (2002) showed that activating AMPARs inhibits NMDARs in a voltage-independent manner. Therefore, blocking AMPARs with NBQX could increase NMDAR ion flux, leading to the increase in synaptic calcium transient amplitude we observed. Alternatively, blocking AMPARs with NBQX may decrease local AMPAR-mediated depolarization thereby increasing the driving force for Ca^2+^, resulting in larger calcium transient amplitudes.

The dendritic calcium transient events that were detected but not analyzed in most of the present study spread outward unidirectionally or bidirectionally along neuronal processes. Variability in spontaneous synaptic calcium transient propagation in dendrites often reflects differences in synaptic spine head and neck geometry, with larger spines more likely to propagate calcium into the dendritic arbor (Noguchi et al., 2005). It is also possible that dendritic calcium transients reflect immature synaptic sites. In DIV 4–5 hippocampal cultures, spontaneous calcium transients described and shown appear more elongated (Andreae and Burrone, 2015) than the synaptic puncta we primarily observe in our ‘mature’ cultures. In the presence of APV, ~14% of synaptic event sites remained active, whereas ~18% of dendritic event sites remained active. This result indicates that NMDARs are crucial for both event subtypes. Dendritic events could correspond to NMDAR upstates. Blockade of these events by extracellular EDTA would confirm this. However, calcium from non-NMDAR sources likely contributes to dendritic events, as evidenced by the fact that these dendritic events responded to APV differently compared to synaptic events: dendritic events maintained similar frequencies to control conditions, but displayed decreased amplitudes compared to controls and PDBu-treated conditions, while synaptic events had decreased frequency compared to controls and no change in amplitude compared to PDBu-treated conditions. Therefore, although NMDARs play a central role in the appearance of most dendritic events and the magnitude of transients, other calcium sources likely also contribute to their appearance as well. Previous studies have exclusively performed synaptic calcium imaging with a cocktail of antagonists, including NBQX, ryanodine, thapsigargin, and nifedipine to block non-NMDAR calcium entry to the dendritic arbor and report strictly NMDAR-dependent calcium transient events (Metzbower et al., 2019; Dean et al., 2022). It could be interesting to test the effects of blocking ryanodine receptors, store-operated calcium entry, or L-type voltage-gated calcium channels individually, rather than all of them indiscriminately, in future studies.

The differences in the effects of NMDAR autoantibodies we observe highlight the increased efficiency and sensitivity of our synaptic calcium imaging pipeline compared to other methods. The first paper characterizing the NMDAR patient antibody 003-102, for example, used a combination of immunocytochemistry to see decreases in NR1-positive clusters, electrophysiology to see decreases in NMDAR-dependent currents, and bath-applied NMDA with somatic calcium imaging to see decreased calcium influx (Kreye et al., 2016). Here, with a single experiment, we can reproduce these findings and extend readouts to synapse function, since we detected fewer active synapses and decreased amplitude of synaptic calcium transients at individual synapses following overnight application of patient antibodies. Changes in amplitude had previously been shown using whole-cell electrophysiology (Kreye et al., 2016; Michalski et al., 2024), which lacks single synapse resolution. In addition, we also found that two patient antibodies (003-102 and 008-218) decreased the frequency of spontaneous calcium transients, a novel finding. Ly et al. (2018) characterized the binding affinities of the NMDAR autoantibodies we tested and found 003-102 and 008-218 had similar binding affinities (with 003-102 formally higher), and 007-124 had the lowest affinity of the three. We found that overnight treatment with antibody 008-218 led to the largest decrease in the number of active synapses, even though it has a similar affinity for NMDARs as 003-102. This could be due to different mechanisms of action. Antibody 003-102 causes NMDAR internalization, whereas 008-218 causes NMDARs to adopt ‘non-active’ conformations where ion channels remain closed (Michalski et al., 2024). This could explain the larger decrease in number of active synapses observed in the presence of 008-218 compared to 003-102. These changes would not be observed as a change in immunostaining using fixed tissue, highlighting the advantage of our assay in testing functional effects of NMDAR autoantibodies on synapses.

Another NMDAR encephalitis antibody has been screened previously with synaptic calcium imaging, although no effect was observed (Dean et al., 2022). It is possible that the treatment time (10 min) was too short, since changes in spontaneous NMDAR current amplitude caused by other NMDAR antibodies occurred only after a 30-min incubation in electrophysiological experiments (Michalski et al., 2024). Dean et al. also used 1 μg/mL of the antibody, whereas our study and others tested concentrations at least an order of magnitude higher (Kreye et al., 2016; Michalski et al., 2024).

The NMDAR encephalitis patient auto-antibodies we tested bind the amino terminal domain of the obligatory GluN1 subunit of NMDA receptors (Ly et al., 2018; Michalski et al., 2024). Given that there are at least eight different subtypes of NMDA receptors that differ in permeability to Ca^2+^, open time, glutamate off-rate (Paoletti et al., 2013), and antigenicity, our pipeline could be used to test effects on specific NMDAR subtypes and subunits. For example, Metzbower et al. reported that a significant portion of spontaneous synaptic calcium transients in hippocampal cultures are driven by GluN2B-containing NMDARs, since the GluN2B-specific blocker ifenprodil reduced amplitude and frequency and silenced ∼30% of synapses. The corticohippocampal cultures we used, have the obligatory GluN1 subunit and GluN2A or GluN2B regulatory subunits, and could be used to test agents that target these subunits. For example, lupus autoantibodies, that potentiate NMDAR function, bind GluN2A and GluN2B subunits (Faust et al., 2010). Populations of synapses with distinct receptor subtypes could potentially be analyzed using our pipeline.

Several research areas could benefit immediately from such a functional synapse assay. NMDAR encephalitis is one such clinically relevant example for which our assay has already shown results. NMDAR encephalitis has an incidence of 1–5 per million people per year (Dalmau et al., 2019); however, it is likely underdiagnosed, with symptoms mimicking other classical psychiatric illnesses (Kayser and Dalmau, 2011). Diagnosis is currently only possible with an abnormal EEG, a spinal tap, and CSF screening with an antibody panel. We observed different effects of the three antibodies we tested, which could potentially underlie three distinct phenotypes of the disease. To better understand and characterize NMDAR encephalitis pathology, a functional antibody screen is essential. Our pipeline could be applied as well to screen patient CSF samples directly, potentially bypassing the need to purify autoantibodies, to directly report if patients show any NMDAR-specific pathologies and thus qualify for antibody-depleting therapies. Our automatic analysis not only avoids manual intervention and potential experimenter selection bias but also allows the detection of more nuanced changes due to the large, unrestricted sample size analyzed.

Our pipeline could also be used to study several conditions known to affect synaptic activity, such as schizophrenia (Glantz and Lewis, 2000; Park et al., 2022), autism spectrum disorder (Monteiro and Feng, 2017), Parkinson’s disease (Reyes-Resina et al., 2024), or Alzheimer’s Disease (Escamilla et al., 2024; Prikhodko et al., 2024). Additionally, this pipeline could be used to investigate how synaptic calcium transients differ between neuronal subtypes, for example, by using different GCaMP6f constructs driven by the CaMKII promoter (for excitatory neurons) or the Dlx promoter (for inhibitory neurons). Sparse transduction could also be used to limit neurite overlap (Metzbower et al., 2019), which would allow synaptic activity from individual neurons to be studied and potential differences between distal and proximal synapses to be observed before and after compound treatments at scale (Walker et al., 2017). GCaMP6f could also be co-expressed with a synapse-targeted fluorophore of a different color to quantify the proportion of total synapses that are active and whether extrasynaptic NMDARs play a role in the calcium transients observed. The pipeline could also be adapted to analyze correlation of spontaneous activity, for example, along individual dendritic branches, between neighboring synapses, or between synapses on different dendrites but innervated by the same presynaptic neuron, possibly in combination with additional genetically encoded fluorophores to mark morphology or cell type. If we assume spontaneously active synaptic populations exist, machine learning models and k-means clustering could be used to classify them. One such framework was recently developed to classify miniature post-synaptic transients from electrophysiological recordings (O’Neill et al., 2025) and could be applied to synapse calcium imaging population analysis.

Although we measure changes in spontaneous synaptic vesicle release, this likely predicts similar changes in evoked release, given that the probabilities of spontaneous and evoked release are correlated at single synaptic sites (Prange and Murphy, 1999). It is possible that our pipeline specifically tests spontaneous synaptic vesicle pools that are distinct from evoked pools (Andreae et al., 2012; Sara et al., 2005). However, other studies have reported that all synaptic vesicles can undergo both spontaneous and evoked fusion (Duan et al., 2024; Hua et al., 2010; Wilhelm et al., 2010).

Our primary goal was to analyze synaptic calcium transients in an automated fashion. With this pipeline, we streamline the analysis of the activity of individual synapses and routinely visualize in a single video what other investigators have used for their entire datasets. In doing so, we open the possibility to study individual synapses on a scale not previously realized. High-throughput large-scale analyses of synapse function could offer new and interesting insights into plasticity and neural network formation *in vitro* and *in vivo*, as well as improve characterization of disease models and development of compounds that prevent or reverse synaptic dysfunction.

## Data Availability

The raw data supporting the conclusions of this article will be made available by the authors without undue reservation.
