# Correction: Automated ROI detection allows rapid quantification of synaptic activity across tens of thousands of synapses in cell culture

**DOI:** 10.3389/fnsyn.2026.1904571

**Published:** 2026-07-20

**Authors:** John Carl Begley, Harald Prüss, Paul Turko, Camin Dean

**Affiliations:** 1 Institute of Biology, Humboldt-Universität zu Berlin, Berlin, Germany; 2 German Center for Neurodegenerative Diseases (DZNE) Berlin, Berlin, Germany; 3 Einstein Center for Neurosciences Berlin, Berlin, Germany; 4 Shared Primary Neuron Facility (SPNF), Institute for Integrative Neuroanatomy, Charité – Universitätsmedizin, Berlin, Germany; 5 Department of Neurology and Experimental Neurology, Charité – Universitätsmedizin Berlin, Berlin, Germany; 6 Bernstein Center for Computational Neuroscience Berlin, Berlin, Germany

**Keywords:** calcium imaging, NMDAR auto-antibodies, automated ROI detection, presynaptic function, post-synaptic function

There was a mistake in Figure 6 as published. The x-axes for the raster plots in Figure 6C are misaligned. The corrected Figure 6 appears below.

**Figure 6:**
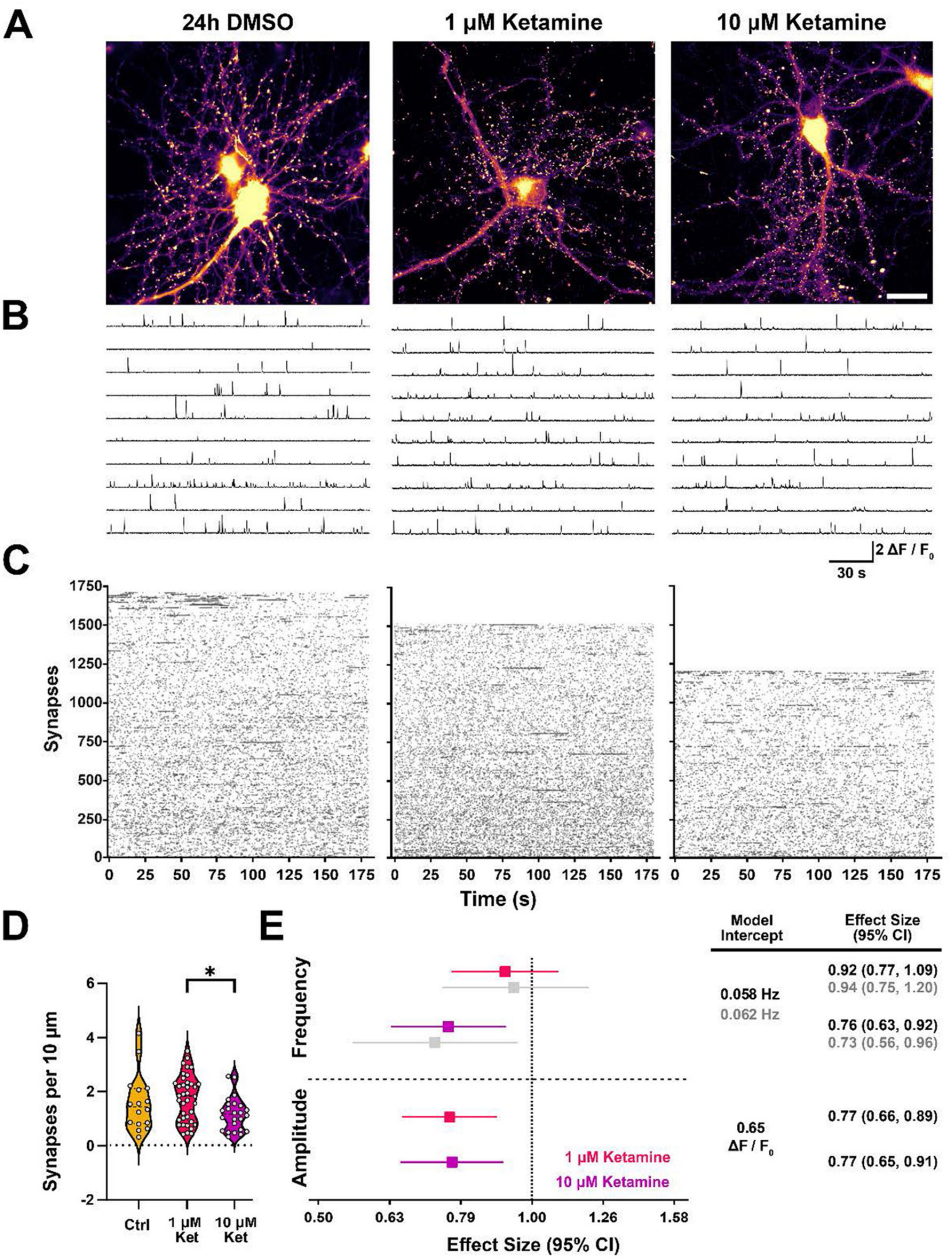
Overnight ketamine treatment reduces the frequency and amplitude of synaptic events. **(A)** Images of maximum fluorescence minus minimum fluorescence projections of 3 min synaptic calcium imaging recordings of regions treated overnight with a DMSO vehicle **(left)**, 1 μm ketamine **(middle)**, or 10 μm ketamine (right; scale bar = 25 μm). **(B)** Example ΔF/F_0_ traces from 10 randomly selected synapses from each condition. **(C)** Raster plots of activity across all synapses in each treatment condition. **(D)** Comparisons of the number of spontaneously active synapses in each condition per 10 μm of GCaMP6f neurite coverage using a Kruskal-Wallis test. **(E)** Forest plot results for synaptic event frequency **(top)** and amplitude **(bottom)** obtained from generalized linear mixed-effects models. 1 μm ketamine (red) and 10 μm ketamine (purple) are shown in reference to 24 h DMSO vehicle controls (vertical dashed line). Bootstrap results for each ketamine treatment for frequency (gray) are shown directly below mixed-effects model results. Model intercepts indicate model-predicted averages for frequency or amplitude in control conditions from mixed-effects models (black) or bootstrap resampling (gray). Effect sizes are reported as mean ± 95% CI, raw values are summarized on the right for mixed-effects models (black) and bootstrap resampling (gray). Model Metrics: Frequency GLMM: ICC = 0.12, Marginal R^2^ = 0.03, Conditional R^2^ = 0.14. Amplitude GLMM: ICC = 0.19, Marginal R^2^ = 0.02, Conditional R^2^ = 0.20. (35 culture preparations; 15–38 regions; DIV 19 and DIV 22). ^*^p < 0.05, ^**^p < 0.01, ^***^p < 0.001, ^****^p < 0.0001.

There was a mistake in Figure 8 as published. The x-axes in Figure 8C were misaligned. The corrected Figure 8 appears below.

**Figure 8:**
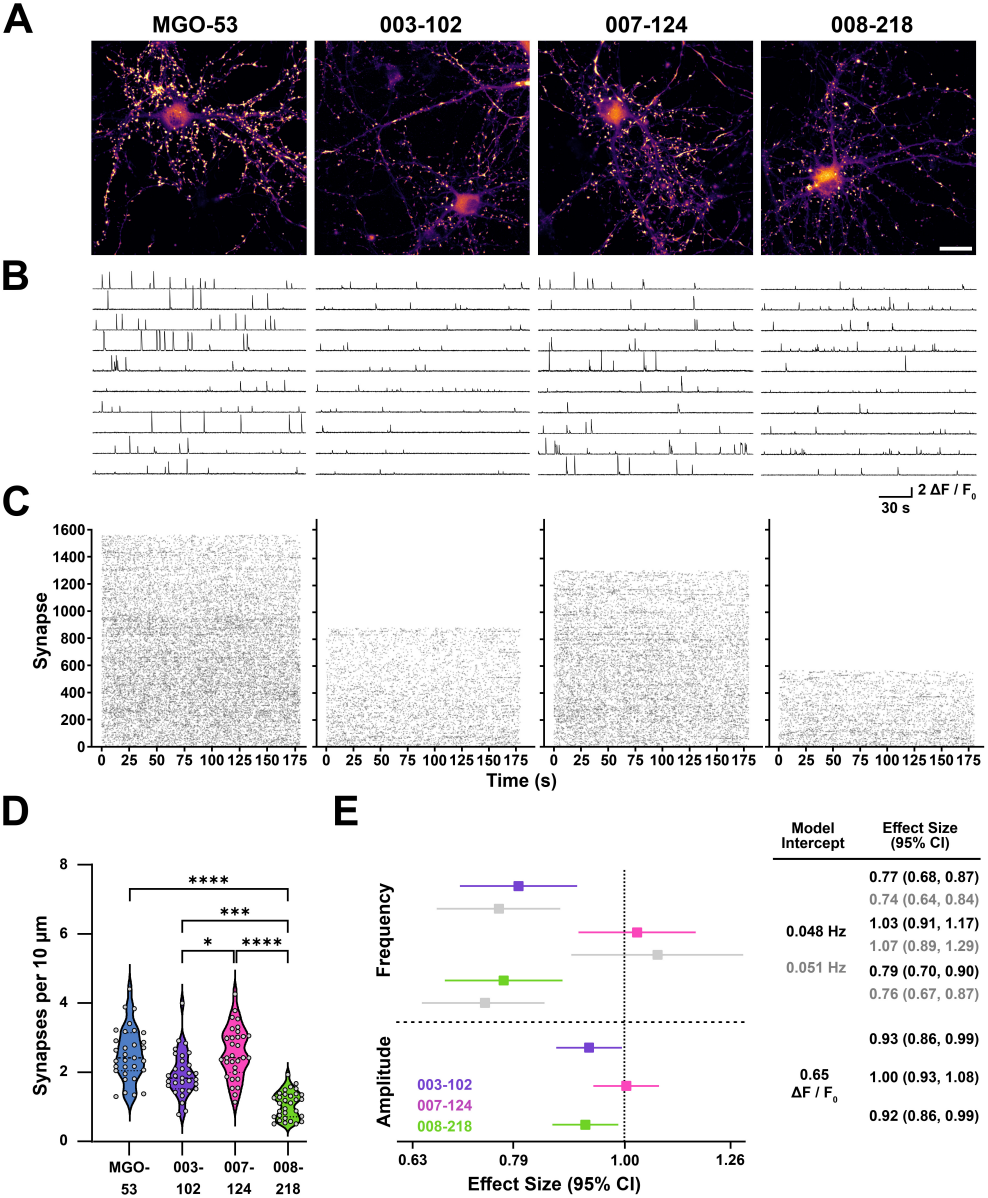
**(A)** Images of maximum fluorescence minus minimum fluorescence projections of 3 min synaptic calcium imaging recordings of regions treated overnight with 15 μg/ml of either control antibody (MGO-53, left), or patient-derived NMDAR autoantibodies (003-102, 007-124, and 008-218; right; scale bar = 25 μm). **(B)** Example ΔF/F_0_ traces from 10 selected synapses from each condition. **(C)** Raster plots of activity across all synapses in each treatment condition. **(D)** Comparisons of the number of spontaneously active synapses in each condition per 10 μm of GCaMP6f neurite coverage using the non-parametric Kruskal-Wallis test. **(E)** Forest plot results for synaptic event frequency (top) and amplitude (bottom) obtained from generalized linear mixed-effects models. Antibody treatments 003-102 (purple), 007-124 (pink), and 008-218 (green) are shown in reference to MGO-53 controls (vertical dashed line). Bootstrap results for each treatment group frequency are shown in gray directly below the mixed-effects model results. Model intercepts indicate model-predicted averages for frequency or amplitude in control conditions from mixed-effects models (black) or bootstrap resampling (gray). Effect sizes are reported as mean ± 95% CI; raw values are summarized on the right for mixed-effects models (black) and bootstrap resampling (gray). Model Metrics: Frequency GLMM: ICC = 0.12, Marginal R^2^ = 0.03, Conditional R^2^ = 0.14. Amplitude GLMM: ICC = 0.06, Marginal R^2^ = 0.005, Conditional R^2^ = 0.06. (4 culture preparations; 15 wells imaged; 31 regions; DIV 20). ^*^p < 0.05, ^**^p < 0.01, ^***^p < 0.001, ^****^p < 0.0001.

A correction has been made to the Figure 8 caption as published. The incorrect sentence for Figure 8A reads as, “**(A)** Images of maximum fluorescence minus minimum fluorescence projections of 3 min synaptic calcium imaging recordings of regions treated overnight with 15 μg/μL of either control antibody (MGO-53, left), or patient-derived NMDAR autoantibodies (003-102, 007-124, and 008-218; right; scale bar = 25 μm).” The correct sentence should read as, “**(A)** Images of maximum fluorescence minus minimum fluorescence projections of 3 min synaptic calcium imaging recordings of regions treated overnight with 15 μg/ml of either control antibody (MGO-53, left), or patient-derived NMDAR autoantibodies (003-102, 007-124, and 008-218; right; scale bar = 25 μm).”

A correction has been made to the **Methods**, *Cell Culture Preparation*, paragraph 1. The incorrect sentence reads as, “A total of 12 litters of rat pups were used (3 for acute treatments with GLYCINE, NBQX, ketamine, memantine, and DMSO vehicle, 4 for anti-NMDAR patient autoantibodies, and 5 for ketamine, memantine, and DMSO vehicle overnight treatments).” The correct sentence should read as, “A total of 12 litters of rat pups were used (3 for acute treatments with glycine, NBQX, ketamine, memantine, and DMSO vehicle, 4 for anti-NMDAR patient autoantibodies, and 5 for ketamine, memantine, and DMSO vehicle overnight treatments).”

A correction has been made to the **Methods**, *Signal Extraction and Normalization*, paragraph 1. The incorrect sentence reads as, “To correct for bleaching and small fluorescence fluctuations, baseline correction was performed using an adaptive iteratively reweighted penalized least squares regression (Zhang et al., 2010).” The correct sentence should read as, “To correct for bleaching and small fluorescence fluctuations, baseline correction was performed using an adaptive iteratively reweighted penalized least squares regression (airPLS; Zhang et al., 2010).”

A correction has been made to the **Results**, paragraph 2. The incorrect sentence reads as, “The raw fluorescence extracted from Suite2p for each ROI (Figure 1F) was then baseline corrected using an automated iterative least squares regression (adaptive iteratively reweighted penalized least squares regression; Zhang et al., 2010) and normalized (ΔF/F0) (Figure 1G).” The correct sentence should read as, “The raw fluorescence extracted from Suite2p for each ROI (Figure 1F) was then baseline corrected using an adaptive iteratively reweighted penalized least squares regression (airPLS; Zhang et al., 2010) and normalized (ΔF/F_0_) (Figure 1G).”

A correction has been made to the **Results**, *AMPA receptors have limited contribution to synaptic calcium transients*, paragraph 2. The incorrect sentence reads as, “This increase likely occurs because more glutamate is available to bind NMDARs in the presence of NBQX.” This sentence should be deleted.

The original version of this article has been updated.

